# Cell-Scale Degradation of Peritumoural Extracellular Matrix Fibre Network and Its Role Within Tissue-Scale Cancer Invasion

**DOI:** 10.1007/s11538-020-00732-z

**Published:** 2020-05-26

**Authors:** Robyn Shuttleworth, Dumitru Trucu

**Affiliations:** Division of Mathematics, University of Dundee, Dundee, DD1 4HN Scotland, UK

**Keywords:** Multiscale modelling, ECM fibre dynamics, Cancer invasion

## Abstract

Local cancer invasion of tissue is a complex, multiscale process which plays an essential role in tumour progression. During the complex interaction between cancer cell population and the extracellular matrix (ECM), of key importance is the role played by both bulk two-scale dynamics of ECM fibres within collective movement of the tumour cells and the multiscale leading edge dynamics driven by proteolytic activity of the matrix-degrading enzymes (MDEs) that are secreted by the cancer cells. As these two multiscale subsystems share and contribute to the same tumour macro-dynamics, in this work we develop further the model introduced in Shuttleworth and Trucu (Bull Math Biol 81:2176–2219, 2019. 10.1007/s11538-019-00598-w) by exploring a new aspect of their interaction that occurs at the cell scale. Specifically, here we will focus on understanding the cell-scale cross talk between the micro-scale parts of these two multiscale subsystems which get to interact directly in the peritumoural region, with immediate consequences both for MDE micro-dynamics occurring at the leading edge of the tumour and for the cell-scale rearrangement of the naturally oriented ECM fibres in the peritumoural region, ultimately influencing the way tumour progresses in the surrounding tissue. To that end, we will propose a new modelling that captures the ECM fibres degradation not only at macro-scale in the bulk of the tumour but also explicitly in the micro-scale neighbourhood of the tumour interface as a consequence of the interactions with molecular fluxes of MDEs that exercise their spatial dynamics at the invasive edge of the tumour.

## Introduction

Cancer cell invasion of tissue is a complex, multiscale process in which gene mutations in healthy cells promote enhanced proliferation and the production of proteolytic enzymes. One of the first steps of the local invasion of tissue is the secretion of matrix-degrading enzymes (MDEs) and the consequential degradation of the extracellular matrix (ECM). The ECM is a non-cellular structure that not only provides support to surrounding cells and tissues, but also acts as a platform for cellular communication. This feature is of particular use to cancer cells, which take advantage of the molecular interactions mediated by various ECM components and favourably utilise these as means for achieving invasion of the surrounding tissue.

The ECM is comprised of a variety of secreted proteins, and the main constituent of the ECM is the structural cross-linked collagen type I, a dense network of fibres that give the ECM its rigidity. In order for a tumour to progress, these strong fibres must be broken down and degraded to free space for the cancer cells. One of the first MDEs to interact with the ECM is the membrane-tethered MMP, MT1-MMP or MMP-14. This MMP exhibits strong collagenolytic capabilities in which they are able to cleave the cross-linked collagen type I fibres and break them into shorter, soluble fibres. These soluble fibres are then degraded by the freely diffusible MMP-2, activated through the cleavage of proMMP-2 molecules by MT1-MMP. This MT1-MMP/MMP-2 cascade is highly effective in promoting tumour invasion through ECM degradation; however, there are some disadvantages to both types of MMPs. MT1-MMP molecules can overcome high-density regions of collagen type I, particularly the cross-linked fibres; however, they do not degrade the collagen, rather cleaving the fibres into smaller fibrils (Tam et al. 2004). On the other hand, MMP-2 cannot degrade the dense cross-linked fibres, but can degrade the smaller, soluble fibrils within the peritumoural region (Van Doren 2015). Consequently, these two MMPs work in harmony with one another for successful invasion of tissue.

Besides the abnormal rate of MDE secretion, the invasive capabilities of a tumour are strengthened by many other processes, including increased proliferation rates and the ability to adapt cellular adhesion properties. Cell adhesion is an essential process in which cells interact and attach to neighbouring cells through calcium-dependent cell-adhesion molecules, known as CAMs, on the cell surface (Humphries et al. 2006). Cell–cell adhesion is dependent on specific cell-signalling pathways that are formed between $\text {Ca}^{2+}$ ions and calcium-sensing receptors in the ECM (Ko et al. 2001). This calcium-dependent cell–cell signalling is regulated by a subfamily of glycoproteins known as E-cadherins that bind with intracellular proteins known as catenins, typically $\beta$-catenin, forming the E-cadherin/catenin complex. Any alteration to the function of $\beta$-catenin will result in a loss of the ability of E-cadherin to initiate cell–cell adhesion (Wijnhoven et al. 2000). This connection between E-cadherin and $\beta$-catenin and the calcium cell-signalling mechanism were first recorded in colon carcinoma (Bhagavathula et al. 2007). Additionally, cells can also bind to the ECM through cell–matrix adhesion (Lodish et al. 2000). Mediated by calcium-independent CAMs, known as integrins, cell–matrix adhesion enables binding of cells to various components of the ECM, such as collagen and fibronectin, with this contributing to cancer cell migration within the tissue. Moreover, enhanced cell–matrix adhesion paired with a loss in cell–cell adhesion facilitates a quicker spread of the cancer cells into the surrounding tissue (Cavallaro and Christofori 2001).

Over the past 25 years, there has been an increasing interest in the mathematical modelling of cancer invasion, see for example (Andasari et al. 2011; Anderson 2005; Anderson et al. 2000; Byrne and Chaplain 1995a, b, 1996; Chaplain et al. 2011, 2006; d’Onofrio 2008; Gerisch and Chaplain 2008; Painter 2008; Painter et al. 2010; Painter and Hillen 2011; Peng et al. 2017; Ramis-Conde et al. 2008; Szymańska et al. 2009; Trucu et al. 2013). Many models of cancer invasion have focussed on the interactions between cancer cells and the extracellular matrix through a variety of different approaches, ranging from continuous (Byrne and Chaplain 1995a, b; Byrne and Preziosi 2003; Painter 2008; Painter et al. 2010) and discrete individual cell-based models (Basanta et al. 2008; Daub and Merks 2013; Hatzikirou et al. 2010; Palm et al. 2016; Tektonidis et al. 2011) to the more complex multiscale modelling approach (Engwer et al. 2016; Kelkel and Surulescu 2012; Kim and Othmer 2013; Peng et al. 2017; Shuttleworth and Trucu 2020, 2019; Stinner et al. 2014; Trucu et al. 2013). Numerous processes of cancer invasion have been addressed, including the effects of proliferation and cellular adhesion (Bitsouni et al. 2017; Byrne and Chaplain 1996; Chauviere et al. 2007; Domschke et al. 2014; Gerisch and Chaplain 2008; Painter 2008; Ramis-Conde et al. 2008), as well as models developed to describe the migration strategies of cancer cells in tissue networks (Chauviere et al. 2007; Hillen 2006; Perumpanani et al. 1998). A model describing both the mesenchymal and amoeboid motion of cells through a fibre network (Painter 2008) concluded that a structured ECM can induce cell aggregation in amoeboid-type cells, whereas the actions of both contact guidance and ECM remodelling are sufficient processes for mesenchymal-type cell invasion to occur.

Numerous processes of cancer invasion have been investigated, including the effects of proliferation and cellular adhesion (Bitsouni et al. 2017; Chauviere et al. 2007; Domschke et al. 2014; Gerisch and Chaplain 2008; Painter 2008; Ramis-Conde et al. 2008; Engwer et al. 2016) as well as the secretion and interaction of proteolytic enzymes, specifically MMPs and uPAs with the ECM (Andasari et al. 2011; Chaplain and Lolas 2005, 2006; Stinner et al. 2014; Peng et al. 2017; Shuttleworth and Trucu 2018; Trucu et al. 2013) during tumour invasion. In particular, a single-scale model developed by Deakin and Chaplain (2013) focussed on the roles of two MMP molecules, namely the membrane-bound MT1-MMP and the soluble MMP-2, where the MT1-MMP/MMP-2 cascade was considered, highlighting the importance of MT1-MMP matrix remodelling within collagen-rich environments. These model dynamics are of great interest to us for the current investigation of the peritumoural MMP processes.

Finally, as the invasion process is naturally multiscale, with its dynamics ranging from subcellular, cellular to tissue scale, major advances have been witnessed in the multiscale modelling of cancer invasion (Anderson et al. 2007; Peng et al. 2017; Ramis-Conde et al. 2008; Shuttleworth and Trucu 2018; Trucu et al. 2013). In particular, advancements have been made in towards two-scale approaches, modelling and appropriately linking the spatiotemporal dynamics occurring at different scales, as first proposed in Trucu et al. (2013) and extended upon in Peng et al. (2017), Shuttleworth and Trucu (2020) and Shuttleworth and Trucu (2019).

In this paper, we aim to advance the novel two-part multiscale modelling framework developed in Shuttleworth and Trucu (2019) and Trucu et al. (2013) by considering the multiscale contribution of ECM fibres within the invasive edge proteolytic dynamics of MDEs. Specifically, by comparison with the framework introduced and developed in Shuttleworth and Trucu (2019) and Trucu et al. (2013), in this work we account for the first time for the direct interaction between the micro-scale dynamics of the peritumoural ECM fibres and the micro-scale activity of the matrix-degrading enzymes, and specifically we focus on the following aspects:

- we explore the enhancing effect that the presence of the ECM fibres has on secretion of the MMP-2 as well as the way the emerging MMP-2 micro-scale spatiotemporal dynamics is influenced by the spatial distribution of ECM micro-fibres within a cell-scale peritumoural neighbourhood of the tumour boundary;
- we account for the micro-scale degradation of the ECM fibres at the tumour interface caused by the MMP-2 peritumoural spatial transport, which has a direct influence upon the continuous micro-scale fibres rearrangement in the peritumoural region, with direct effects upon the macro-scale tumour dynamics both in terms of its evolving spatial morphology and in terms of the associated cell–cell and cell–matrix adhesion processes;
- we address these important interactions both in the context of a single cancer cells population and in the context of two cancer cells populations (which considers both a primary and a mutated cells population).

This will pave the way for the establishment of an additional cell-scale link between the peritumoural micro-scale fibres rearrangements and the micro-scale proteolytic MDEs activities at the tumour boundary, which ultimately extends and complements the two-part multiscale framework introduced in Shuttleworth and Trucu (2019) where the linking of its two constituent multiscale systems was so far mediated only through the shared macro-dynamics.

## Brief Modelling Overview of the Multiscale Fibre Dynamics on the Topological Closure of the Tumour

As this work builds upon the two-part multiscale model introduced and developed in Shuttleworth and Trucu (2019) that investigates cancer invasion within a heterogeneous ECM, let us start by revisiting the multi-component structure of the ECM and its multiscale interaction witch the cancer cell population as considered in Shuttleworth and Trucu (2019). To that end, we devote this section to recapitulate the key details of the framework terminology that we introduced in Shuttleworth and Trucu (2019) and Trucu et al. (2013), with special focus in the following three subsections on recasting the multiscale fibres dynamics within cancer invasion occurring on the topological closure of the expanding tumour.

Let us start by denoting the support of the invading tumour region by $\varOmega (t)$ and assume this evolves within the maximal reference tissue cube $Y \in \mathbb {R}^N$ for $N=2,3$, centred at the origin of the space. At any spatio-temporal point $(x,t) \in \varOmega (t) \times [0,T]$, we consider the tumour to be comprised of a cancer cell population *c*(*x*, *t*), integrated within a multiphase heterogeneous ECM density denoted by *v*(*x*, *t*). Specifically, the heterogeneous ECM is regarded as comprising of a *fibres* component and a *non-fibre* soluble component. We denote the tissue-scale (macro-scale) density of a *non-fibre* ECM phase by *l*(*x*, *t*), and we consider this to include all non-fibrous components of the ECM, i.e. elastin, laminins, fibroblasts, etc. On the other hand, the macro-scale mass density of the *fibres* ECM phase is denoted by *F*(*x*, *t*) and accounts for all fibrous proteins such as collagen and fibronectin within the matrix.

For completion, in the following subsections, we will briefly revisit the key points about the ECM fibres multiscale structure and dynamics as well as its interactive contribution within the overall spatiotemporal tumour evolution.

### The Multiscale ECM Fibre Structure and Its Contribution to the Tissue Dynamics

**Fig. 1:**
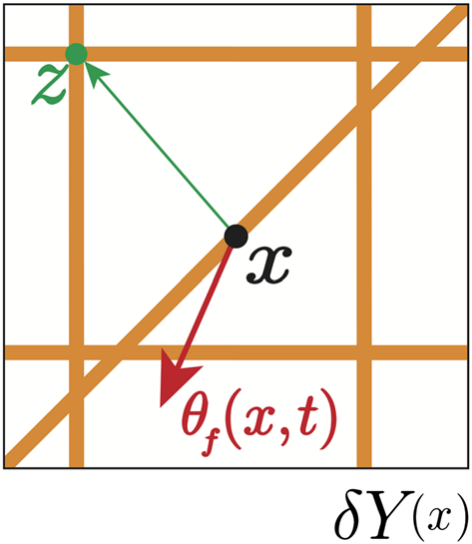
Schematic of the micro-fibres distribution on the micro-domain $\delta Y(x)$, centred at *x*, with the barycentral position vector $\overrightarrow{x \,z}:=z-x$ pointing towards an arbitrary micro-location $z\in \delta Y(x)$ illustrated by the green arrow (Color figure online)

As derived in Shuttleworth and Trucu (2019), at any macro-position $x \in \varOmega (t_{0})$, the fibrous ECM phase can be represented through a macro-scale vector field, denoted $\theta _{f}(x,t)$, that captures not only the macroscopic distribution of fibres *F*(*x*, *t*), but also their naturally arising macroscopic orientation that is induced by their mass distribution of micro-fibres, denoted *f*(*z*, *t*), over a given micro-domain $\delta Y(x)$ centred at the given macroscopic point *x* of cell-scale $\delta >0$ (i.e. $z\in \delta Y(x)$ and $\delta Y(x):=x+\delta Y$). An example of micro-fibres *f*(*z*, *t*) patterns over a micro-domain $\delta Y(x)$ (defined in “Appendix B”), alongside the naturally emerging macroscopic fibre orientation $\theta _{f}(x,t)$ that was derived in Shuttleworth and Trucu (2019), is shown schematically in Fig. 1.

In brief, while referring to its full derivation presented in Shuttleworth and Trucu (2019), the naturally generated revolving barycentral orientation $\theta _{{f,\delta Y(x)}}(x,t)$ associated with $\delta Y(x)$ is given by the *Bochner mean value* of the position vectors function

$$\begin{aligned} \delta Y(x)\ni z\longmapsto z-x\in \mathbb {R}^{N} \end{aligned}$$

with respect to the density measure $f(z,t)\lambda (\cdot )$, where $\lambda (\cdot )$ is the usual Lebesgue measure (see Yosida 1980), and so this is expressed mathematically as:

1 $$\begin{aligned} \theta _{{f,\delta Y(x)}}(x,t)=\frac{\int \limits _{\delta Y(x)}f(z,t)(z-x)\mathrm{d}z}{\int \limits _{\delta Y(x)} f(z,t) \mathrm{d}z}. \end{aligned}$$

Following on, at any spatio-temporal point (*x*, *t*), this revolving barycentral orientation $\theta _{{f,\delta Y(x)}}(x,t)$ induces the naturally arising macroscopic fibre orientation vector field representation that is defined as

2 $$\begin{aligned} \theta _{{f}}(x,t)=\frac{1}{\lambda (\delta Y(x))} \int _{\delta Y(x)} f(z,t) \ \mathrm{d}z \cdot \frac{\theta _{{f,\delta Y(x)}} (x,t)}{||\theta _{{f,\delta Y(x)}} (x,t)||}. \end{aligned}$$

Furthermore, as observed in (2), the macroscopic mean-value fibre representation at any (*x*, *t*) is given by the Euclidean magnitude of $\theta _{{f}}(x,t)$, namely,

3 $$\begin{aligned} F(x,t):=||\theta _{{f}}(x,t)||_{2}. \end{aligned}$$

Thus, the total ECM distributed at any spatiotemporal point (*x*, *t*) is therefore given by $v(x,t)=l(x,t)+F(x,t)$.

### Macro-scale Dynamics

For the tissue dynamics of a tumour consisting of a single-cell population, as in Shuttleworth and Trucu (2019), we denote by $\mathbf{u} (x,t)$ the macro-scale *global tumour vector*, consisting of the cancer cells population and the fibre and non-fibre components of the surrounding two-phase ECM, namely

$$\begin{aligned} \mathbf{u} (x,t)=(c(x,t),F(x,t),l(x,t)). \end{aligned}$$

Further, the tumour’s *volume of occupied space* is given as

$$\begin{aligned} \rho (\mathbf{u} (x,t)) = \vartheta _{v}(F(x,t)+l(x,t)) + \vartheta _{c}c(x,t), \end{aligned}$$

where $\vartheta _{v}$ and $\vartheta _{c}$ represent the fractions of physical space occupied by the entire ECM and the cancer cells, respectively.

Focussing first on the cancer cell population, the spatial movement of the cancer cells is governed by random motility (approximated here by diffusion) and a cell-adhesion process that includes both cell–cell and cell–matrix adhesion, with cell–matrix adhesion accounting for both cell–fibre and cell–non-fibre adhesion. Assuming the cells are subject to a logistic proliferation law, the dynamics of the cancer cell population can be mathematically represented as

4 $$\begin{aligned} \frac{\partial c}{\partial t} = \nabla \cdot [D_{1} \nabla c - c \mathcal {A}(x,t,\mathbf{u} (\cdot ,t),\theta _{f}(\cdot ,t))] + \mu _{1}c(1 - \rho (\mathbf{u} )) \end{aligned}$$

where $D_{1}$ and $\mu _{1}$ are the non-negative diffusion and proliferation rates, respectively. The nonlocal adhesive flux $\mathcal {A}(x,t,\mathbf{u} (\cdot ,t),\theta _{f}(\cdot ,t))$ accounts for the bias induced in the spatial movement of the cancer cells due to cellular adhesion properties between each other (cell–cell adhesion) and the surrounding environment (cell–matrix adhesion accounting for both cell–fibre and cell–non-fibre adhesion). Specifically, the adhesive flux $\mathcal {A}(x,t,\mathbf{u} (\cdot ,t),\theta _{f}(\cdot ,t))$ appearing in (4) considers the interactions of cancer cells within a *sensing radiusR* with the other cancer cells and two-phase ECM distributed on the *sensing region*$\mathbf {B}(x,R):=x+\{\xi \in \mathbb {R}^{N}\,|\, \parallel \xi \parallel _{{2}}<R\}$ and is described by the following nonlocal term

5 $$\begin{aligned} \mathcal {A}(t,x,\mathbf{u} (\cdot , t), \theta _{{f}}(\cdot , t))= & {} \frac{1}{R} \int _{\mathbf {B}(0,R)} \mathcal {K}(\parallel \xi \parallel _{2}) \big (n(\xi ) (\mathbf{S} _{{cc}} c(x+\xi ,t) + \mathbf{S} _{{cl}} l(x+\xi ,t)) \nonumber \\&\quad + \hat{n}(\xi ) \ \mathbf{S} _{{cF}} F(x+\xi ,t) \big )(1-\rho (\mathbf{u} ))^{+} \end{aligned}$$

Moreover, the influence of the spatially distributed adhesive interactions on the sensing region $\mathbf{B} (x,R)$ is accounted through a radial kernel $\mathcal {K}(\cdot ):\mathbf {B}(0,R)\rightarrow \mathbb {R}$ which is taken here to be of the form

6 $$\begin{aligned} \mathcal {K}(\xi ):=\frac{2\pi R^2}{3}\left( 1-\frac{\parallel \xi \parallel }{2R}\right) , \quad \xi \in \mathbf {B}(0,R) . \end{aligned}$$

Furthermore, the strength of the bonds created between the cells distributed at *x* and the other cells or non-fibrous ECM phase distributed at $(x+\xi )\in \mathbf {B}(x,R)$ in the direction of the unit normal

7 $$\begin{aligned} n(\xi ):= \left\{ \begin{array}{ll} \xi /||\xi ||_2 &{}\quad \text {if} \ \xi \in \mathbf {B}(0,R){\setminus }\{(0,0)\}, \\ (0,0) &{}\quad \text {if} \ \xi =(0,0). \end{array} \right. \end{aligned}$$

is explored through the cell–cell and cell–non-fibrous ECM adhesion strength coefficients $\mathbf{S} _{cc}$ and $\mathbf{S} _{cl}$, respectively. We consider $\mathbf{S} _{cl}$ to be constant, while the coefficient representing cell–cell adhesion, $\mathbf{S} _{cc}$, is monotonically dependent on the levels of extracellular $\text {Ca}^{2+}$ ions which enable robust adhesive bonds between neighbouring cells (Gu et al. 2014; Hofer et al. 2000). Therefore, we assume $\mathbf{S} _{cc}$ is dependent on the underlying non-fibrous ECM phase smoothly ranging from 0 to a $\text {Ca}^{2+}$-saturation level denoted $\mathbf{S} _{{\max }}$ and is taken as

$$\begin{aligned} \mathbf{S} _{{cc}}(x,t):=\mathbf{S} _{{max}}e^{\big ({1-\frac{1}{1-(1-l(x,t))^2}}\big )}. \end{aligned}$$

The last term in (5) considers the interactions between the cancer cells and the fibrous ECM phase distributed within the region $\mathbf{B} (x,R)$. The strength of this interaction is proportional to the distribution of macro-fibres at $F(x+\xi ,t)$ and it is the orientation of these fibres, $\theta _{f}(x+\xi ,t)$, that biases the direction in which adhesive bonds are formed in the direction $\hat{n}(\cdot )$

8 $$\begin{aligned} \hat{n}(\xi ):= \left\{ \begin{array}{ll} \frac{\xi +\theta _f(x+\xi )}{||y+\theta _f(x+y)||_{{2}}}, &{}\quad \text {if} \ (\xi +\theta _f(x+\xi )) \ne (0,0), \\ (0,0) \in \mathbb {R}^2, &{}\quad \text {otherwise}. \end{array} \right. \end{aligned}$$

Finally, the macroscopic dynamics of the ECM involving both the fibrous and non-fibrous phase on the invading tumour domain $\varOmega (t)$ is dictated by an overall degradation by the cancer cells of both the fibrous and non-fibrous ECM phases and is described mathematically as

9 $$\begin{aligned} \frac{\mathrm{d} F}{\mathrm{d} t}&= -\gamma _{1} cF, \end{aligned}$$

10 $$\begin{aligned} \frac{\mathrm{d} l}{\mathrm{d} t}&= -\gamma _{2} cl, \end{aligned}$$

where $\gamma _{1}, \gamma _{2}$ are the degradation rates for fibres and non-fibres ECM phases, respectively.

The coupled macro-scale dynamics expressed mathematically in (4) and (9)–(10) takes place in the presence of zero Neumann boundary conditions, and with the initial conditions

11 $$\begin{aligned} c(0,x):=c_{0}(x), \quad F(0,x):=F_{0}(x), \quad \text { and } \quad l(0,x):=l_{0}(x), \quad \forall x\in Y, \end{aligned}$$

which will be detailed in Sects. 5.2 and 6 as appropriate for the considered computational cases.

### Microscopic Fibre Rearrangement Instigated by the Macro-scale Cell Flux

As derived in Shuttleworth and Trucu (2019), during the macro-dynamics, as the cancer cells invade the surrounding tissue, they have the ability to push the fibres in the direction of their spatial flux and rearrange the micro-fibre mass distributions, thereby reorienting the macro-fibres direction. Thus, at time *t* and at any spatial location $x \in \varOmega (t_{0})$, the micro-fibres $f(z,t), \ \forall z \in \delta Y(x)$ undergo a microscopic rearrangement process fuelled by the cancer cells spatial flux given mathematically as

$$\begin{aligned} \mathcal {F}(x,t) := D_{1} \nabla c(x,t) - c(x,t) \mathcal {A}(x,t,\mathbf{u} (\cdot ,t),\theta _{f}(\cdot ,t)). \end{aligned}$$

As a direct consequence, the micro-fibres $f(z,t), \ \forall z \in \delta Y(x)$ are acted upon uniformly by a *rearrangement flux vector*

$$\begin{aligned} r(\delta Y(x),t):=\omega (x,t)\mathcal {F}(x,t)+(1-\omega (x,t))\theta _{f}(x,t), \end{aligned}$$

which is triggered by the spatial flux of the cancer cells $\mathcal {F}(x,t)$ that is balanced in a weighted manner by the oriented vector field $\theta _{f}(x,t)$ of the existing distribution of fibres at (*x*, *t*) and the amount of cells exercising spatial transport at (*x*, *t*), with a naturally emerging weight given by

$$\begin{aligned} \omega (x,t)=\frac{c(x,t)}{c(x,t)+F(x,t)}. \end{aligned}$$

Further, as detailed in Shuttleworth and Trucu (2019), under the influence of the rearrangement flux vector, $r(\delta Y(x),t)$, an appropriate level of micro-fibres mass *f*(*z*, *t*) will undergo a spatial movement towards a new position

$$\begin{aligned} z^{*}:=z + \nu _{{\delta Y(x)}}(z,t) \end{aligned}$$

where the relocation direction and magnitude are given by

12 $$\begin{aligned} \nu _{{\delta Y(x)}}(z,t)=\left( x_\text {dir}(z) + r(\delta Y(x), t)\right) \cdot \frac{f(z,t)(f_{\max }-f(z,t))}{f^{*}+||r(\delta Y(x)) - x_\text {dir}(z)||_{2}} \cdot \chi _{{\{f(\cdot ,t)>0\}}}{,} \end{aligned}$$

with $f_{\max }$ representing the maximum amount of micro-fibres at $z\in \delta Y(x)$, and the relative degree of fibre’s occupancy at *z* being denoted by $f^{*}(z,t):=\frac{f(z,t)}{f_\text {max}}$. Finally, the spatial transport of micro-fibres at *z* will be exercised in accordance with the physical space available at the new position $z^{*}$, which is explored through a movement probability $p_{move}:=\max (0,1-f^{*}(z^{*},t))$. Specifically, an amount of micro-fibres $p_{move}f(z,t)$ will be moved to the new position $z^{*}$, while the rest of the micro-fibres will remain at *z*.

## Novel Modelling for the MDE Proteolytic Micro-scale Dynamics at the Tumour Interface

To incorporate the dynamics of peritumoural micro-scale fibres within the MDE micro-dynamics at the tumour invasive edge, let us start by exploring the influence of the macro-fibre distributions on the emergence of a cell-scale molecular source of MDE at the tumour interface. This will effectively enhance the *top-down* macro–micro-link derived and introduced in Trucu et al. (2013) (which was considered in Shuttleworth and Trucu (2019)) for the boundary micro-dynamics and in return will influence the *bottom-up* feedback link to macro-dynamics. Finally, MDEs micro-dynamics occurring within a cell-scale neighbourhood of the tumour boundary will be explored on an appropriately selected covering bundle of boundary micro-domains $\{\epsilon Y\}_{\epsilon Y \in \mathcal {P}}$, which was introduced and constructed with complete details in Trucu et al. (2013).

*The top-down link* As discussed previously, the expansion of the tumour boundary is dependent on the peritumoural degradation of ECM by the matrix-degrading enzymes (MDEs). However, the secretion of MDEs induced by the distribution of cancer cells in the outer proliferating rim is dependent upon the structure of the ECM and in particular upon the fibre distribution. Indeed, in the presence of a high distribution of ECM fibres, the cancer cells exhibit a strong rate of MT1-MMP secretion, which in turn leads to an increase in the activation of proMMP-2 molecules (Zigrino et al. 2001). Thus, since in this emerging MT1-MMP/MMP-2 cascade, the secretion rate of MT1-MMP correlates directly with the amount of MMP-2 molecules, we obtain therefore that the presence of the ECM fibres enhances the production of the MMP-2 molecules that are then released at the tumour boundary.

Thus, on any microscopic time interval initiated at $t_{0}\in [0,T]$, namely on $[t_{0}, t_{0}+\varDelta t]$, with $\varDelta t>0$, the MMPs source at each microscopic location $y \in \epsilon Y \cap \varOmega (t_{0})$ and any microscopic time $\tau \in [0,\varDelta t]$ arises as a collective contribution of the cancer cells within the outer proliferating rim which are further enhanced by the presence of ECM fibres. Therefore, at any $(y, \tau )\in \epsilon Y \times [0, \varDelta t]$ the MMPs source can be mathematically expressed as

13 $$\begin{aligned} 1. \quad&g_{\epsilon Y}(y,\tau ) = \frac{\int \limits _\mathbf{B (y,\gamma )\cap \varOmega (t_0)} \alpha c (x,t_0 + \tau ) \ \widetilde{F}(x,t_0 + \tau ) \ \mathrm{d}x}{\widetilde{F} \cdot \lambda (\mathbf{B} (y,\gamma )\cap \varOmega (t_0))}, \quad y \in \epsilon Y \cap \varOmega (t_0), \nonumber \\ 2. \quad&g_{\epsilon Y}(y,\tau ) = 0,\quad y \in \epsilon Y {\setminus } \big ( \varOmega (t_0)+\{ \zeta \in Y| \ ||\zeta ||_2 < \gamma \}), \end{aligned}$$

where $\gamma$ represents the maximum thickness of the outer proliferating rim, $\mathbf {B}(y,\gamma ):=\{\zeta \in \mathbb {R}^{N}\,|\, \parallel y-\zeta \parallel _{{\infty }}\le \gamma \}$ and $\alpha$ is an MMP secretion rate for the cancer cell population. Furthermore, the function $\widetilde{F}(x,t + \tau ):=1+F(x,t)$, $\forall \, t>0$ explores the spatially distributed enhancement of the source of MMPs produced by the cancer cells that is enabled through the presence of fibres (Zigrino et al. 2001) (i.e. a higher distribution of fibres inducing a greater number of MMP-2 molecules). Finally, $\widetilde{F} \cdot \lambda$ represents the underlying fibre density measure that is defined by

$$\begin{aligned} \widetilde{F} \cdot \lambda (G):=\int \limits _{G} \widetilde{F}(x, {t_{0}+\tau }) \ \mathrm{d}x, \quad G \in \varSigma (Y), \end{aligned}$$

where $\lambda$ is the standard Lebesgue measure on $\mathbb {R}^{2}$, and *G* is a nonempty Borel subset set of *Y*, i.e. $G\in \varSigma (Y)$, with $\varSigma (Y)$ representing the Borel $\sigma$-algebra on *Y*.

In the presence of this source, on any micro-domain $\epsilon Y$, a cross-interface MMP-2 diffusive transport takes place, and as the MMP-2 finds it easier to exercise their random movement in regions of lower micro-fibres density, the diffusion rate is micro-fibre density dependent, and therefore this spatiotemporal micro-dynamics can be mathematically formulated as

14 $$\begin{aligned} \frac{\partial m}{\partial \tau } = \underbrace{D_m{(\tilde{f})} \varDelta m}_{\text {diffusion}} \ \ +\underbrace{g_{\epsilon Y}(y,\tau )}_{\text {source term}}, \quad y \in \epsilon Y, \quad \tau \in [0,\varDelta t] \end{aligned}$$

with the fibre-dependent diffusion coefficient

$$\begin{aligned} D_m{(\tilde{f)}}=\frac{D}{1+\alpha _{m} \tilde{f}(y,t_{0}+\tau )}, \end{aligned}$$

where *D* is the baseline diffusion rate and $\alpha _{m}$ being a *“slowing down” constant factor* induced by the presence of the micro-fibres density $\tilde{f}(y,t_{0}+\tau )$, which is defined as follows. For this, let us observe first that for the two separate bundles of micro-domains, namely:

- the bundle of boundary micro-domain $\{\epsilon Y\}_{\epsilon Y\in \mathcal {P}(t_{0})}$ that captures only the MMP-2 micro-dynamics,
- the bundle of fibres micro-domains $\{\delta Y(x)\}_{x\in \{x_{i,j}\}_{i,j=1,q}}$ (as illustrated in Fig. 3);

we have that these two families get to overlap over the peritumoural region (as illustrated in Fig. 3). Thus, we have that for a given location *y* within our boundary micro-domain $\epsilon Y$, there is the possibility to have more than one fibre micro-domains $\delta Y(x)$ that covers the location *y*. Therefore, we obtain this way a micro-scale set-valued mapping that surveys the number of points *z* corresponding to up to four-fibre micro-domains $\delta Y(x)$ that cover the same micro-position $y\in \epsilon Y$, namely

15 $$\begin{aligned} \begin{array}{cl} &{}\epsilon Y\ni y\longmapsto z(y)\in \bigcup \limits _{x\in Y} \delta Y(x)\\ \text { is given by: }\\ z(y) \,\,\,\text { is } &{} \text { the set of the micro-spatial points within up to four } \delta Y(x)(y), \\ \text { where }&{}\\ &{} \text {each selected } \delta Y(x)(y) \text { is a micro-cube within } \bigcup \limits _{x\in Y} \delta Y(x) \\ &{} \text { with the property that this contains } y\in \epsilon Y \\ &{} \text { (please see Fig.~}3 \text { for an illustration of this situation) }. \end{array} \end{aligned}$$

Therefore, we have that

16 $$\begin{aligned} \tilde{f}(y,t_{0}+\tau ):= \left\{ \begin{array}{lll} f(z(y),t_{0}+\tau ) &{}\quad \text {if} &{} card(z(y))=1;\\ \frac{1}{card(z(y))}\sum \limits _{\zeta \in z(y)}f(\zeta , t_{0}+\tau ) &{}\quad \text {if} &{} card(z(y))>1. \end{array} \right. \end{aligned}$$

Finally, as for the micro-dynamics, we assume that the bundle of micro-domains $\{\epsilon Y\}_{\epsilon Y\in \mathcal {P}(t)}$ covers the entire cell-scale interfacial region of molecular activity of MMP-2; the micro-dynamics (14) takes place in the presence of zero-flux boundary conditions. Furthermore, as we consider that there is no pre-existing distribution of MMP-2 on $\epsilon Y$ (before the emergence of the MMP-2 molecular micro-dynamics), we assume therefore that the e micro-dynamics (14) is initiated with zero initial conditions, i.e. $m(y,0):=0$, $\forall y\in \epsilon Y$.

*The bottom-up link* During their micro-dynamics, the MMPs diffuse into the surrounding ECM and it is the pattern of their advancing spatial distribution that controls the degradation of the peritumoural ECM captured within each micro-domain $\epsilon Y$. This degradation ultimately leads to the movement of the tumour boundary, whereby a movement direction $\eta _{\epsilon Y}$ and displacement magnitude $\xi _{\epsilon Y}$ are derived from the pattern of ECM degradation in each micro-cube $\epsilon Y$ (and for full derivation, we refer the reader to Trucu et al. (2013)). The microscopic movement of the boundary is represented at the macro-scale through the movement of the boundary midpoint $x^{*}_{\epsilon Y}$ to a new spatial position $\widetilde{x^{*}_{\epsilon Y}}$. Thus, although the *bottom-up* link of the model is much akin to previous works (Shuttleworth and Trucu 2018, 2019), the specific context in which the presence of micro-fibres distribution enables on the one hand an enhanced source of MMP and on the other hand acts as an impediment for their random motility, the MMP micro-dynamics within the peritumoural region of the tumour domain $\varOmega (t_{0})$ incorporates now these important aspects, ultimately resulting in an improved estimate for the macroscopic boundary movement characteristics (i.e. encapsulated by the movement direction $\eta _{\epsilon Y}$ and displacement magnitude $\xi _{\epsilon Y}$ ). This crucial micro-scale-induced boundary relocation is then translated back to the macro-scale, resulting in an expanded tumour domain $\varOmega (t_{0}+\varDelta t)$ on which the multiscale dynamics is continued (as detailed in Trucu et al. (2013)).

### Microscopic Fibre Degradation

Given the proteolytic properties of the MMPs, the cell-scale cross-interface MMP-2 micro-dynamics will result in a direct peritumoural ECM degradation, whose pattern not only depends on the amount of MMP-2 transported at a given location *y* within a given micro-domain $\epsilon Y$, but also on the existing ECM micro-fibres spatial distribution at *y*. To address this micro-scales interaction that takes place between the MMP-2 cross-interface transport and micro-fibres within any given micro-domain $\epsilon Y$, we consider that the spatial patterns of micro-fibres distributions that are aligned with the MMP-2 flux $\nabla m(\cdot , t_{0}+\tau )$ suffer less degradation than those that are positioned orthogonal to it. Thus, for any fibre micro-domain $\delta Y(x)$ that has nonempty intersection with $\epsilon Y$, by denoting $\varPhi _{fm}(\cdot ,\cdot ):\delta Y(x)\times [0,\varDelta t]\rightarrow [0,\pi ]$ the function that explores these emerging angles, given by

17 $$\begin{aligned} \varPhi _{fm}(z,\tau )= \left\{ \begin{array}{lll} \arccos \left( \frac{<\nabla m(z,\tau ) , \nabla f(z,t_{0}+\tau )>}{||\nabla m(z,\tau )||_{{2}} || \nabla f(z,t_{0}+\tau )||_{{2}}}\right) \chi _{{\delta Y(x)\,\cap \,\epsilon Y}}(z), &{}\quad if &{}\quad \parallel \nabla f(z, t_{0}+\tau ) \parallel _{{2}}>0, \\ \frac{\pi }{2} \chi _{{\delta Y(x)\,\cap \, \epsilon Y}}(z), &{}\quad if &{}\quad \parallel \nabla f(z, t_{0}+\tau ) \parallel _{{2}}=0. \end{array} \right. \end{aligned}$$

where $\chi _{{\delta Y(x)\,\cap \, \epsilon Y}}(\cdot )$ is the usual characteristic function of $\delta Y(x)\cap \epsilon Y$.

The strength of the micro-fibre degradation rate is influenced by their degree of alignment with the flux of the MMPs, which is explored by the angle $\varPhi _{fm}(z,\tau )$ at which the flux of the MMP-2 molecules acts upon the mass distribution of micro-fibres $f(z, t_{0}+\tau )$ at each micro-position *z*, and so this can therefore be mathematically formalised as

18 $$\begin{aligned} D_{g}(z,\tau ):= d_{f} \cdot \exp \left( 1-\frac{1}{1 - (1 - \varPhi (z,\tau ))^2}\right) . \end{aligned}$$

where $d_{f}$ is a nonnegative degradation constant, and the function $\varPhi (\cdot ,\cdot )$

$$\begin{aligned} \varPhi (z,\tau ) = \left| \frac{\varPhi _{fm}(z,\tau )}{\varPhi _{\text {max}}}\right| , \end{aligned}$$

represents the influence of the collision angle relative to the maximum angle $\varPhi _{\text {max}}:=\frac{\pi }{2}$ where the highest rate of degradation will occur (i.e. when the flux of MMPs is perpendicular upon the micro-fibres mass). Thus, the micro-fibre dynamics at location *z* in the $\delta$-sized micro-scale can be mathematically represented as

19 $$\begin{aligned} \frac{\partial f}{\partial \tau } = - D_{g}(z,\tau ) \cdot f(z,{t_{0}+}\tau ), \qquad z \in \delta Y, \ \tau \in [0,\varDelta t]. \end{aligned}$$

where the initial condition at microscopic time $\tau =0$ is given by the pre-existing distribution of micro-fibres $f(z,t_{0})$.

## Brief Summary of the Macro-scale and Interface Proteolytic Micro-scale Dynamics of the New Multiscale Moving-Boundary Modelling Framework

The tissue- and cell-scale boundary dynamics of the new multiscale moving-boundary model proposed here can therefore be summarised as:

20a $$\begin{aligned} \text {Macro-scale}&\,\,\text { dynamics:} \nonumber \\ \frac{\partial c}{\partial t}&= \nabla \cdot [D_{1} \nabla c - c \mathcal {A}(t,x,\mathbf{u} (\cdot ,t), \theta _{{f}}(\cdot , t))] +\mu _{1}c(1-\rho (\mathbf{u} )), \nonumber \\ \frac{\mathrm{d} F}{\mathrm{d} t}&= -\gamma _{1} c F , \nonumber \\ \frac{\mathrm{d} l}{\mathrm{d} t}&= -\gamma _{2} c l + \omega (1-\rho (\mathbf{u} )), \end{aligned}$$

20b $$\begin{aligned} \text {Micro-scale}&\,\,\text {leading-edge MMP-2-dynamics within peritumoural micro-fibres:}\nonumber \\ \frac{\partial m}{\partial \tau }&= D_{m} {(f)}\varDelta m +g_{\epsilon Y}(y,\tau ), \quad y \in \epsilon Y, \ \tau \in [0,\varDelta t], \nonumber \\ \frac{\partial f}{\partial \tau }&= - D_{g} \cdot f(z,{t_{0}+}\tau ), \quad z \in \delta Y, \ \tau \in [0,\varDelta t]. \end{aligned}$$

**Fig. 2:**
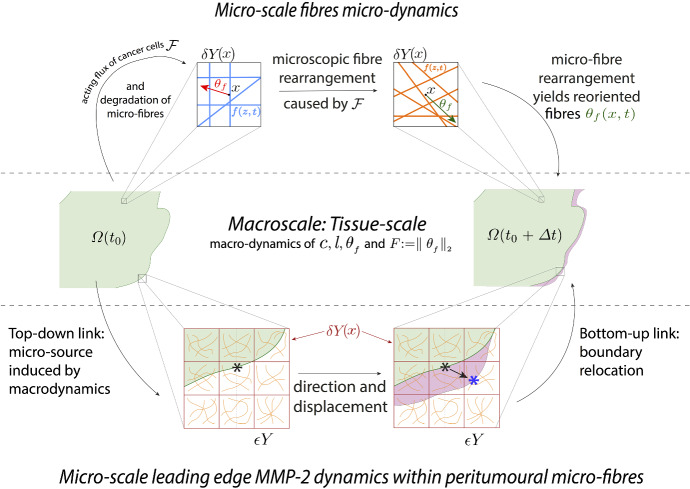
Schematic illustrating the novel global multiscale framework (Color figure online)

The macro- and leading-edge MMP-2 micro-dynamics summarised in (20) take forward and enhance the multiscale moving-boundary framework introduced earlier in Shuttleworth and Trucu (2019) leading to a new extended framework that incorporates two interconnected multiscale systems, which is schematically represented in Fig. 2. These two multiscale systems share the same macro-scale tissue-level dynamics (summarised in (20a)) and at the same time capture the interactions between two micro-scale systems that are different in nature that but which are linked to the two macro-dynamics through two double feedback loops.

While the macro-scale tissue dynamics describe the evolution of the spatial distribution of cancer cells and both the non-fibres and fibres ECM phase, the micro-scale part of the first multiscale system governs the dynamic rearrangement of fibres. Specifically, the rearrangement of the fibres emerges as a consequence of the acting macro-scale flux of the cancer cell spatial flux upon the mass distribution of micro-fibres distributed within micro-domains $\delta Y(x)$ which are centred at any given macroscopic point $x\in Y$. The redistributed micro-fibres naturally yield a new macroscopic vector field representation of the newly oriented fibres that will have a cascade influence upon adhesion processes the cancer cell population tissue scale dynamics biasing their migration, as detailed in Shuttleworth and Trucu (2019).

The second multiscale system involved in this modelling brings into the picture contribution of the micro-scale proteolytic dynamics occurring in a cell-scale neighbourhood of the tumour interface. Here, we take forward the multiscale moving-boundary approach initially introduced in Trucu et al. (2013) and recapitulated in Shuttleworth and Trucu (2019), by considering the influence that the oriented macro-scale ECM fibres has upon the emergence of the micro-scale MMP-2 source and at the same time exploring the explicit cell-scale interaction that takes place between the MMP-2 spatial flux and the mass distribution of micro-fibres at micro-scale which results in fibre degradation, summarised in (20b), notably triggering changes in the macroscopic fibres orientation. Consequently, these leading-edge MMPs dynamics instigate a change in the position of the tissue-scale tumour boundary that corresponds to the pattern of peritumoural ECM degradation, this way allowing the macro-dynamics to continue on the newly enlarged tumour region and thus the invasion process continues.

## Numerical Approaches and Initial Conditions for Computations

Expanding on the multiscale moving-boundary framework developed in Shuttleworth and Trucu (2019) building on the model initially introduced in Trucu et al. (2013), we developed a new modelling and finite differences computational approach to address specifically the cell-scale peritumoural interaction between the MDE and the micro-fibres mass distributions at micro-scale. Specifically, we explore the link between fibre distribution and MDE density at the tumour interface, in addition to the MDE induced micro-fibre degradation at the cell scale.

### Brief Description of the Numerical Approach

To address the tumour macro-dynamics, we use the novel predictor–corrector method developed and fully defined in Shuttleworth and Trucu (2019) that accounts for the complexity of the cancer dynamics. For this, we consider a uniform spatial mesh of size $h=0.03125$, and we use a combination of central differences and mid-point methods to discretise the local spatial operators, while involving an off-grid approach (introduced and detailed in Shuttleworth and Trucu (2019)) for the calculation of the non-local adhesion terms (that enable the adhesive flux) at each spatiotemporal node.

**Fig. 3:**
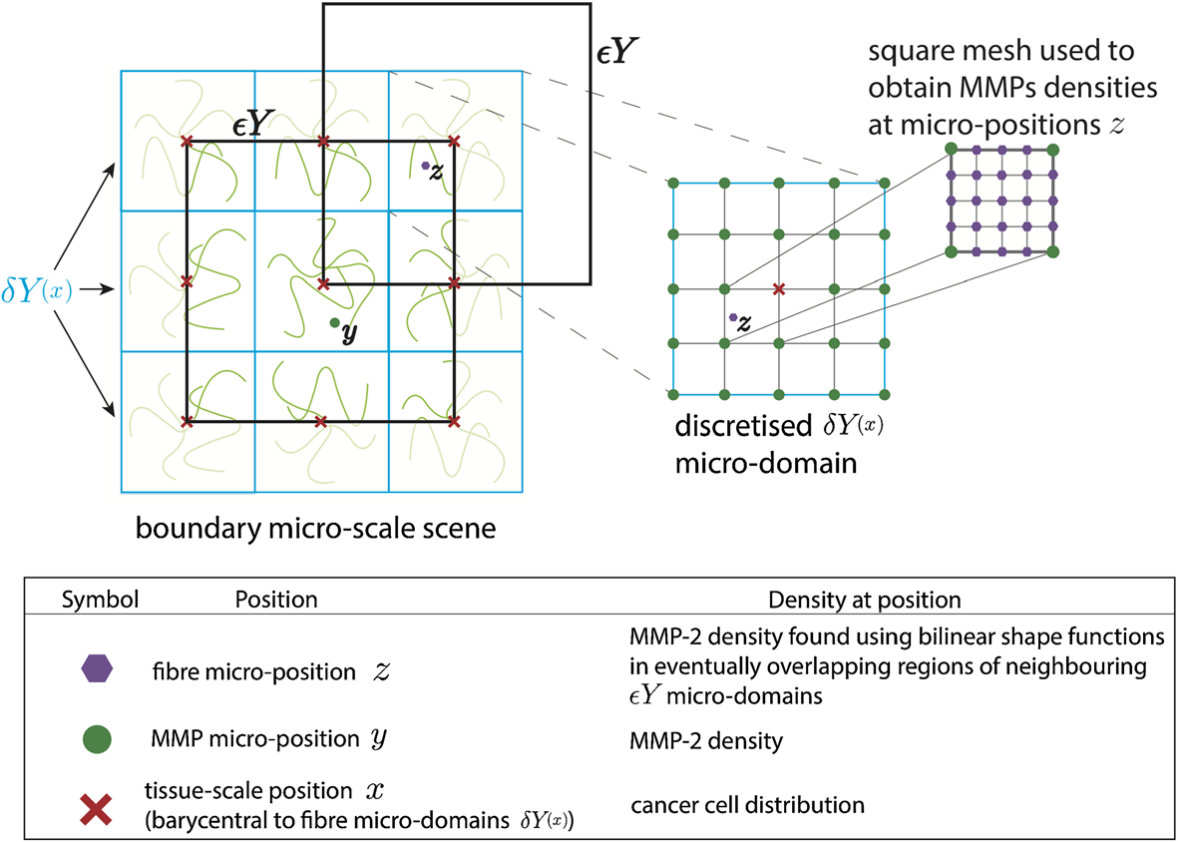
Schematic illustrating the boundary micro-scales computational setting where the micro-dynamics is explored

Furthermore, to obtain the microscopic boundary relocation described in Shuttleworth and Trucu (2018), we explore the *top-down* and *bottom-up* link, using a finite difference approach for computing the MDE micro-dynamics occurring on the bundle of boundary micro-domains $\{\epsilon Y\}_{\epsilon Y\in \mathcal {P}(t_{0})}$ over a micro-scale time span $[0,\varDelta t]$ corresponding to the macro-scale time interval $[t_{0},t_{0}+\varDelta t]$, where $\varDelta t>0$ is an appropriate time length. Hence, while considering a time discretisation of the micro-scale time span $[0,\varDelta t]$ into *p* uniformly distributed micro-time steps of size $\delta t := \frac{\varDelta t}{p}$, each boundary micro-cube $\epsilon Y$ is also discretised uniformly with a $q\times q$ square grid with equally distributed of spatial mesh of size $h_{\epsilon }:=\frac{\epsilon }{q-1}$, i.e. $\varDelta y_{{1}} = \varDelta y_{{2}} = h_{\epsilon }$. Therefore, to discretise the reaction-diffusion equation (14), we start by addressing the spatially discretised source term induced by the cancer cell and fibre distribution (13) in the way it was described in Trucu et al. (2013). Then, to solve the spatiotemporal dynamics in (14), we develop a similar predictor–corrector method using explicit Euler predictor and trapezoidal corrector for the time marching, while for the spatial operators involved we use again a combination of mid-points and central differences, which in this context are given by

21 $$\begin{aligned} \begin{aligned} \nabla \cdot [\nabla m]_{i,j}^{n}&= \text {div}[\nabla m]^{n}_{i,j} \\&\simeq \frac{[m_{y_{{2}}}]^{n}_{i+\frac{1}{2},j} - [m_{y_{{2}}}]^{n}_{i-\frac{1}{2},j}}{\varDelta y_{{2}}} + \frac{[m_{y_{{1}}}]^{n}_{i,j+\frac{1}{2}} - [m_{y_{{1}}}]^{n}_{i,j-\frac{1}{2}}}{\varDelta y_{{1}}} \end{aligned} \end{aligned}$$

wherewith $n = 0, \ldots , p$ and $i,j = 1, \ldots , q$, where $p, q\in \mathbb {N}{\setminus }\{0,1\}$. Finally, important for capturing the degradation of the mass distribution of peritumoural micro-fibres at micro-scale, we use bilinear shape functions on square elements (Hughes 1987) to appropriately interpolate the solutions and the associated fluxes of the MMP-2 micro-dynamics in the eventually overlapping regions of their $\epsilon Y$s boundary micro-domains. This way the appropriate MMP-2 flux information is obtained at any micro-position *z* in any given intersecting $\delta Y(x)$ fibres micro-domains, i.e. within those fibres micro-domains $\delta Y (x)$ in the peritumoural region for which

$$\begin{aligned} \text { there exists a } \epsilon Y \text { micro-domain such that } \delta Y (x)\cap \epsilon Y\ne \emptyset . \end{aligned}$$

as illustrated in Fig. 3.

All the simulations of the model included in this paper have been developed in MATLAB.

### Initial Conditions Used in Computational Simulations

**Fig. 4:**
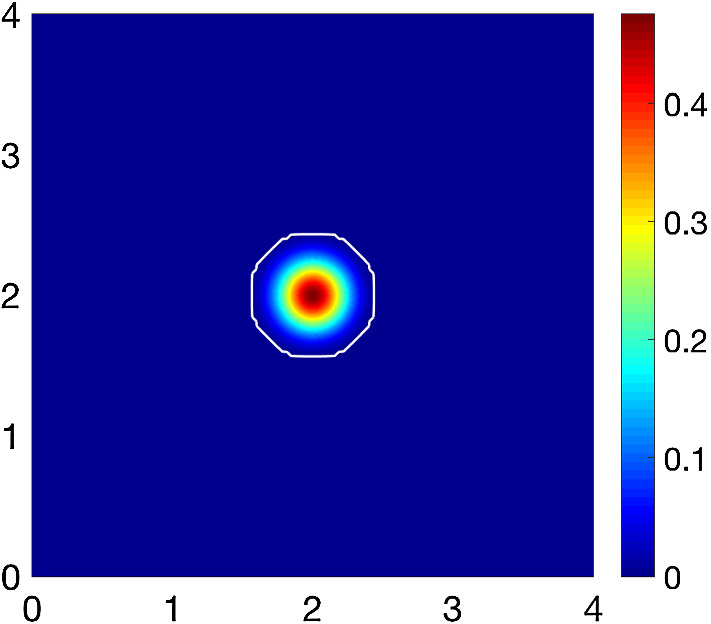
Initial condition for the cancer cell population *c*(*x*, 0), illustrating the tumour boundary by the white contour

We consider the same initial conditions from (Shuttleworth and Trucu 2019), such that we consider the initial cancer cell population $c(x,0):=c_{0}(x)$ to occupy the region $\varOmega (0) = \mathbf{B} ((2,2),0.5)$ positioned in the centre of the 2D tissue domain $Y:=[0,4]\times [0,4]$, as shown in Fig. 4,

22 $$\begin{aligned} c_{0}(x)=0.5\left( \text {exp}\left( -\frac{||x-(2,2)||^2_2}{0.03}\right) -\text {exp}(-28.125)\right) \left( \chi _{{\mathbf {B}((2,2),0.5-\gamma )}} *\psi _{\gamma }\right) , \end{aligned}$$

where $\psi$ is the standard mollifier detailed in “Appendix A” that acts within a radius $\gamma<<\frac{\varDelta x}{3}$ from $\partial \mathbf{B} ((2,2),0.5-\gamma )$ to smooth out the characteristic function $\chi _{{\mathbf {B}((2,2),0.5-\gamma )}}$.

We explore the invasion of cancer within two different compositions of the non-fibrous ECM phase, a homogeneous distribution given as

23 $$\begin{aligned} l_{0}(x)=\text {min}\{0.5,1-c(x,0)\} \end{aligned}$$

and a heterogeneous distribution, previously explored in Shuttleworth and Trucu (2019) and defined below

24 $$\begin{aligned} l(x,0)=\text {min}\left\{ h(x_1,x_2), 1- c(x,0)\right\} , \end{aligned}$$

where for any $x:=(x_{1},x_{2}) \in Y\text { and } \zeta = 7\pi$ we have

$$\begin{aligned} h(x)&=\frac{1}{2}+\frac{1}{4}\text {sin}(\zeta y_1(x) y_2(x))^3 \cdot \text {sin}(\zeta \frac{y_2(x)}{y_1(x)}), \\ with:\quad&\\ y_{1}(x)&:= \frac{1}{3}(x_{1}+1.5), \\ y_{2}(x)&:= \frac{1}{3}(x_{2}+1.5). \end{aligned}$$

**Fig. 5:**
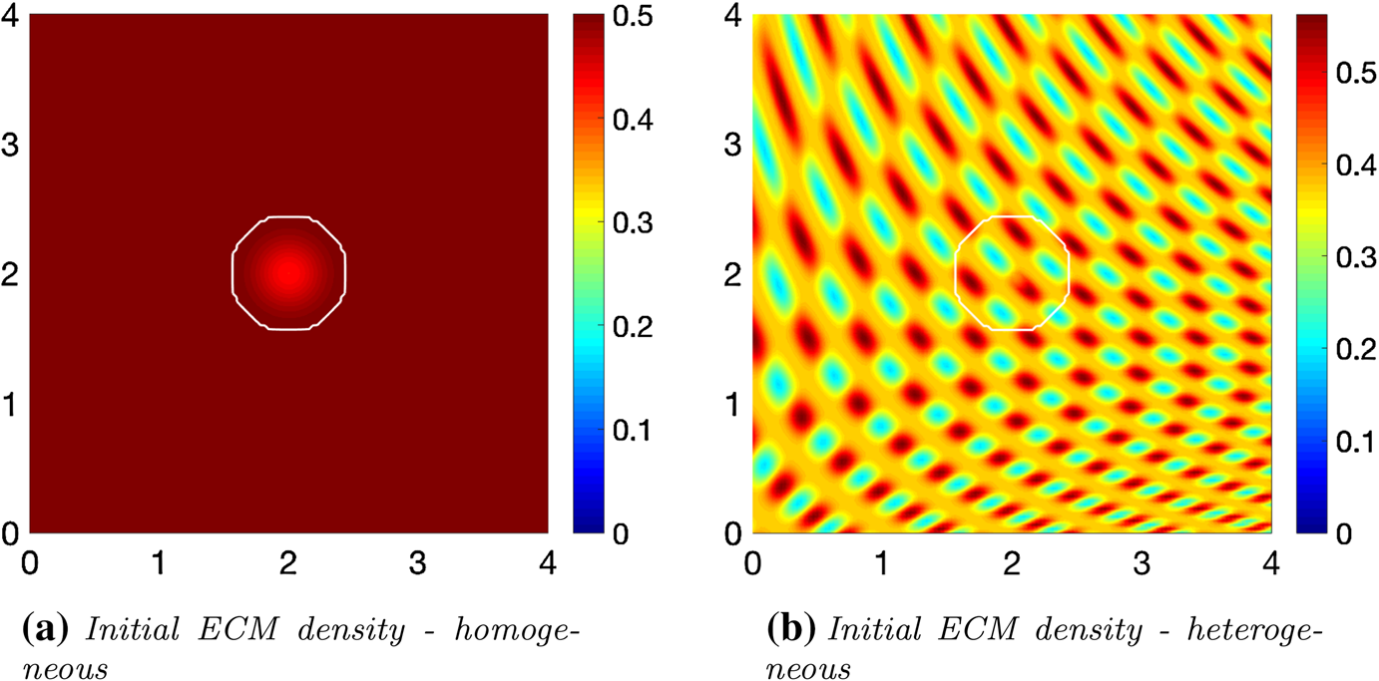
Initial conditions for the non-fibres ECM phase *l*(*x*, 0), illustrating both a homogeneous (**a**) and heterogeneous (**b**) distribution

These initial conditions are shown in Fig. 5.

Finally, the fibrous ECM phase will be initialised with both a homogeneous and heterogeneous macro-scale distribution. To this end, we first assume a random distribution of five pre-assigned micro-distributions containing patterns of five different micro-fibres (detailed in “Appendix B”) that are then randomly assigned onto $\delta Y(x) := x + \delta Y$. To calibrate the macro-fibre distributions, we use the initial conditions for the non-fibrous ECM phase, for either a homogeneous or heterogeneous distribution, (23) and (24), respectively, taking a percentage *p* of the density of the non-fibres ECM at each spatiotemporal position *x*. This allows for the control of the maximal height of the micro-fibres in each $\delta Y(x)$, centred at macro-position *x*, so that the resulting macro-fibre initial distribution $F_{0}(x)$ represents the percentage *p*, which here will be $15\%, \ p=0.15$ or $20\%,\ p=0.2$, with the latter representing a denser collagen structure.

## Results

**Fig. 6:**
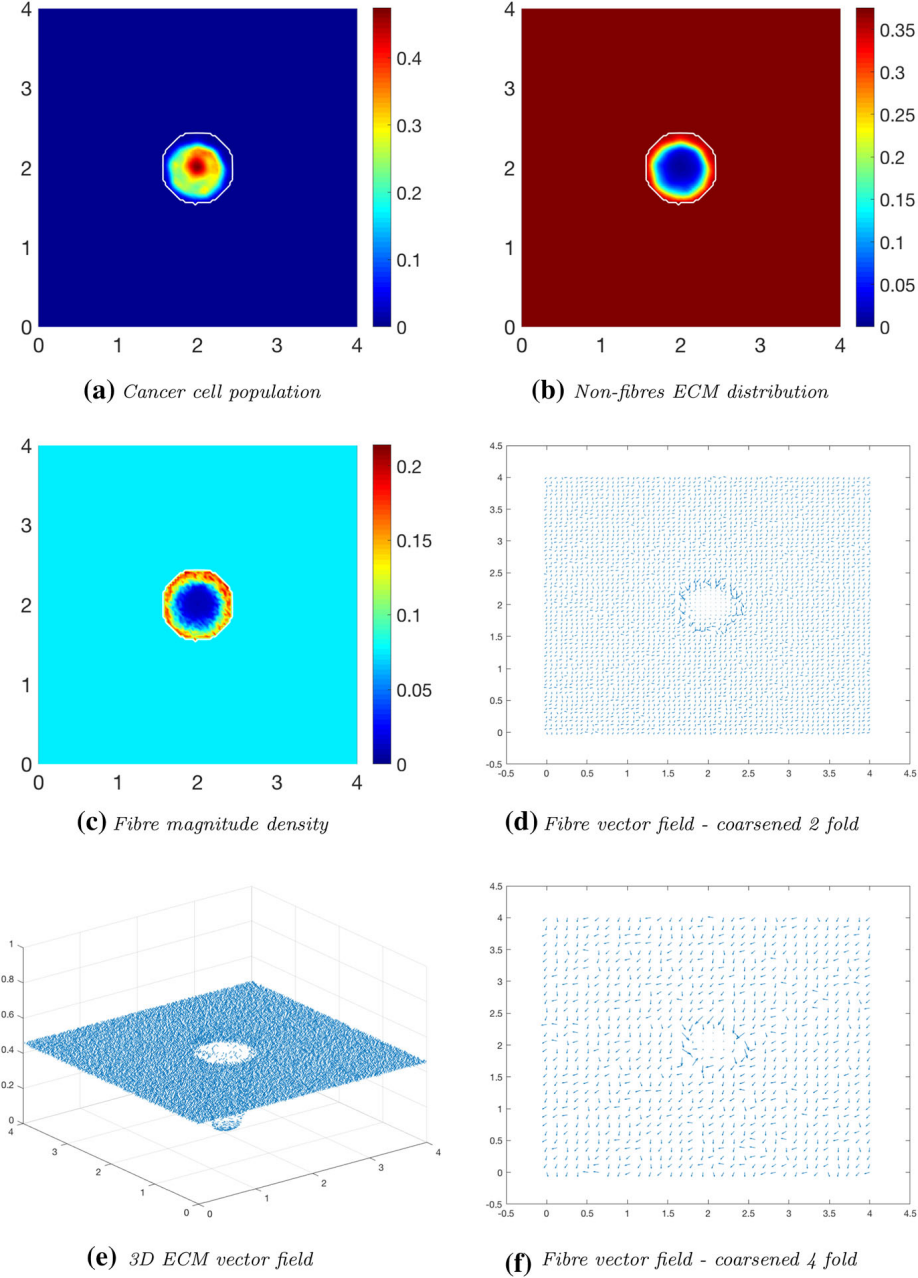
Simulations at time $25\varDelta t$ with a homogeneous distribution of the non-fibrous and fibres phase of the ECM and a micro-fibres degradation rate of $d_f = 1$

**Fig. 7:**
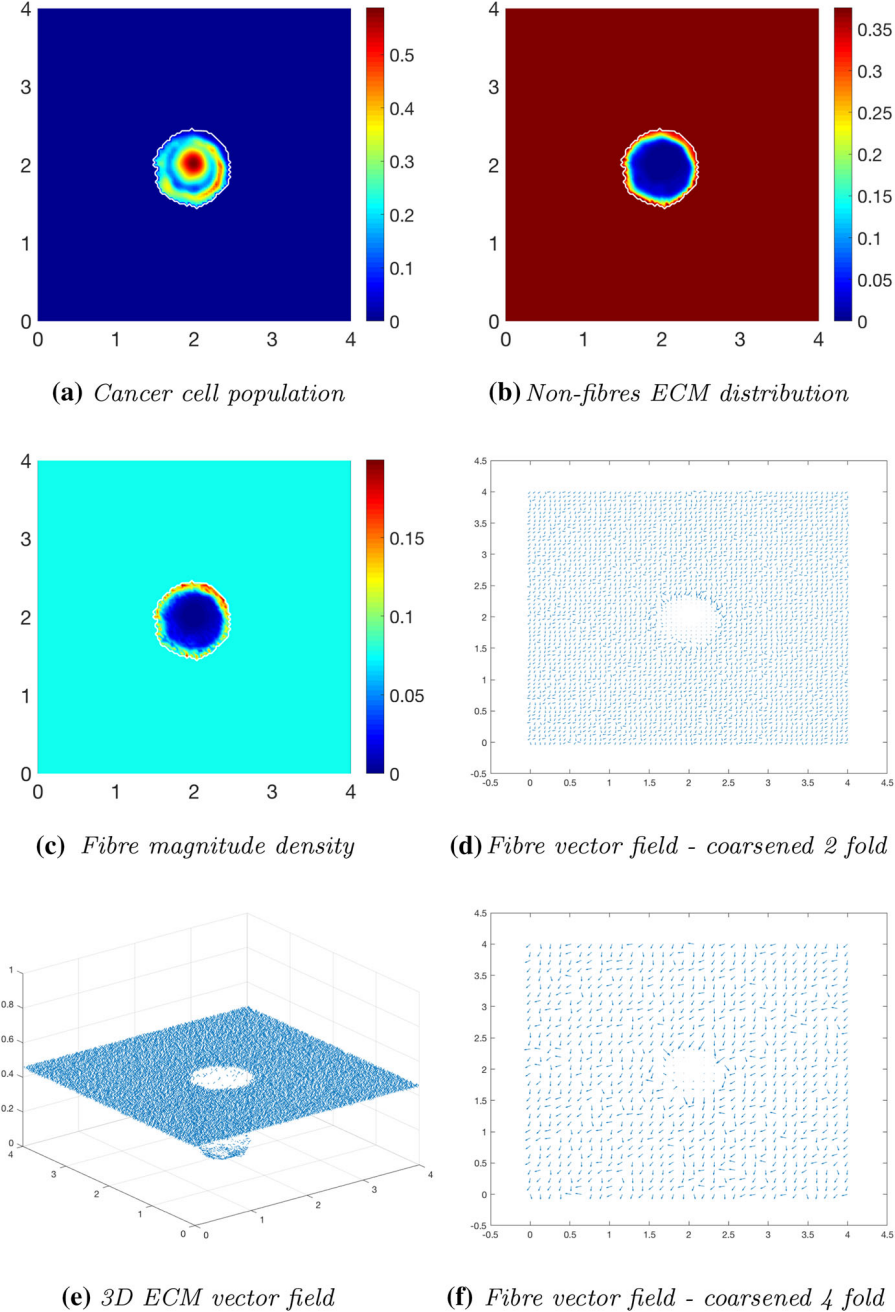
Simulations at time $50\varDelta t$ with a homogeneous distribution of the non-fibrous and fibrous phase of the ECM and a micro-fibres degradation rate of $d_f = 1$

**Fig. 8:**
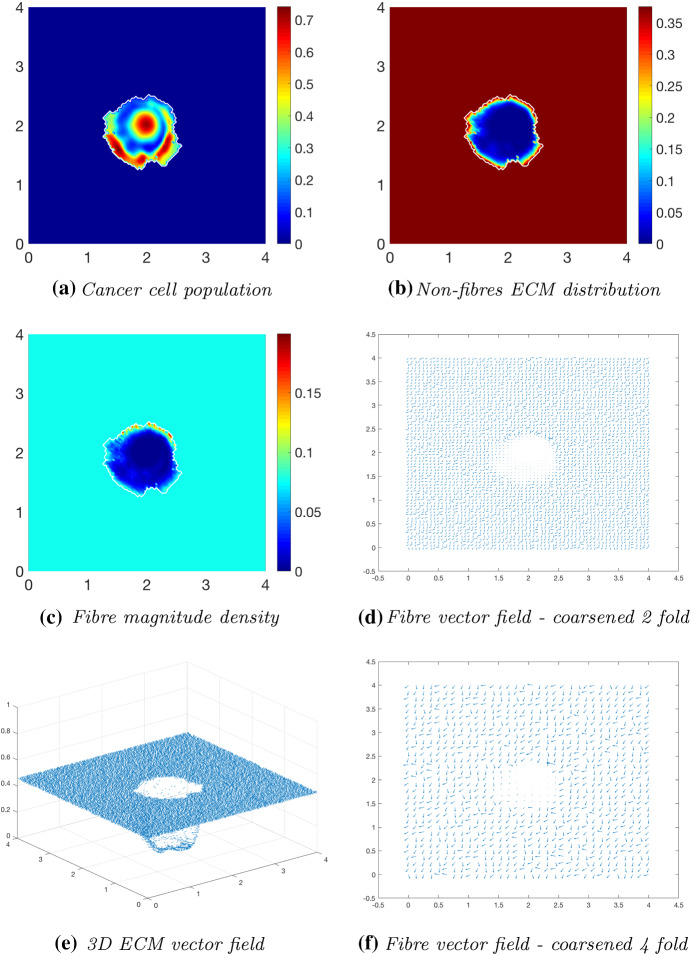
Simulations at time $75\varDelta t$ with a homogeneous distribution of the non-fibrous and fibrous phase of the ECM and a micro-fibres degradation rate of $d_f = 1$

We first explore tumour invasion in the presence of a homogeneous ECM, where the non-fibres phase is the homogeneous distribution (23) with the fibres phase taking the percentage $p=0.15$ of *l*(*x*, 0). Using the parameter set $\varSigma _{1}$ from “Appendix C”, the microscopic fibre degradation rate $d_f=1$, and the cell adhesion coefficients

$$\begin{aligned} \mathbf{S} _{max}=0.5, \quad \mathbf{S} _{cF}=0.1 \quad \text {and} \quad \mathbf{S} _{cl}=0.05, \end{aligned}$$

we investigate the effects micro-fibres degradation on tumour progression. Figures 6, 7 and 8 display the evolution of the tumour at times $25 \varDelta t$, $50\varDelta t$ and $75\varDelta t$, respectively. Each figure contains the subfigures: (a) cancer cell population; (b) non-fibres ECM density; (c) macroscopic fibre distribution; vector fields of fibre orientations at two different resolutions, namely: (d) coarsened twofold; and (f) coarsened fourfold ; and finally in (e) a 3D plot of the orientation of the complete ECM distribution $v(x,t)=F(x,t)+l(x,t)$.

After $25 \varDelta t$ macro-stages, as shown in Fig. 6, the boundary of the tumour has remained largely unchanged, as shown in Fig. 6a. Both the non-fibrous and fibrous ECM phases undergo degradation where the cancer cell distribution is highest (Fig. 6b, c), while minor degradation of the fibres has also occurred at the tumour boundary. The masses of micro-fibres have been rearranged such that the macroscopic orientation of fibres remains in line with the general fibre direction (Fig. 6d, f) which points towards the origin of the space. However, there are some irregularities, particularly visible in Fig. 6f which has been magnified fourfold, where the orientation of the fibres is mixed with some fibres realigned to near perpendicular of their original orientation. The cancer cells are pushing and rearranging the fibres in a direction opposite to that of the initial fibre orientation, as shown in Fig. 6c, where a build-up of fibre distributions occurs on the top right of the tumour region, situated close to the bulk of the tumour mass. As the tumour expands in size, the fibres are subsequently degraded during each stage of evolution, as shown in Figs. 7 and 8, illustrating the simulations after $50 \varDelta t$ and $75 \varDelta t$, respectively. At time $50\varDelta t$, some cells have detached and formed a region encircling the initial bulk of tumour cells (Fig. 7a). The boundary of the tumour is expanding into the surrounding tissue (Fig. 7b), exhibiting a “rippling” effect along the proliferating edge caused by the fibre-mediated movement of the boundary. The macroscopic fibre distribution becomes depressed as the cancer cells increase in distribution and thus increase in their degradative behaviour (Fig. 7c). Moving on to final time $75 \varDelta t$, the tumour has largely increased in size and there are dense regions of cell distribution (Fig. 8a). The cells are gathering at the tumour interface, pulled in this direction by cell–fibre adhesion, following the direction of the fibres observed at the previous interval in Fig. 7d, f. The source of MMPs induced by the cancer cells is very high in these dense areas; therefore, the degradation of fibres is higher (Fig. 8c) witnessed here by the absence of macroscopic fibre distribution.

**Fig. 9:**
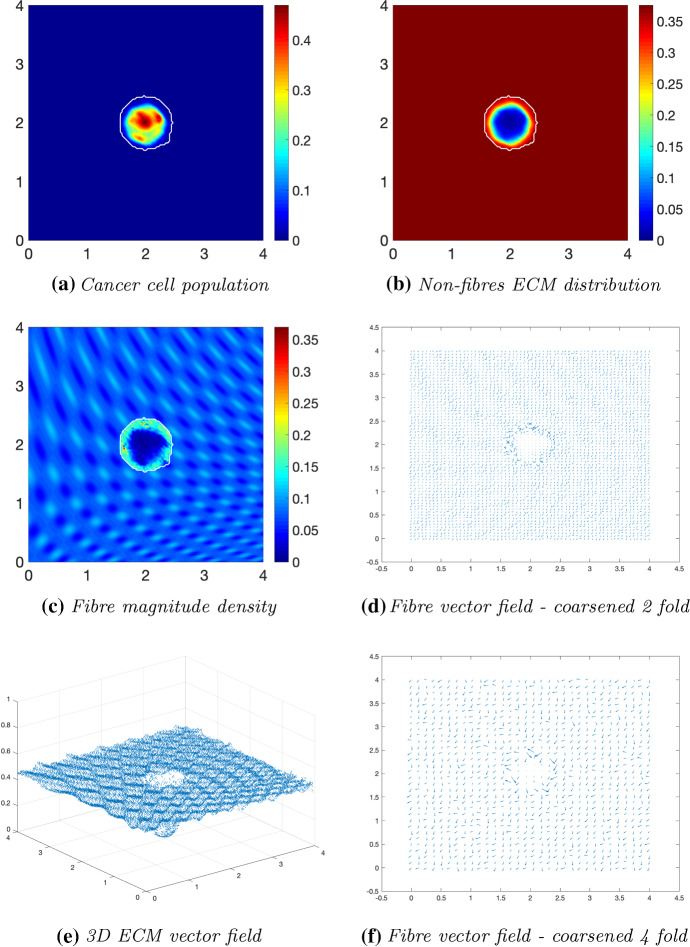
Simulations at time $25\varDelta t$ with a homogeneous distribution of the non-fibrous phase and $15\%$ heterogeneous fibres phase of the ECM with a micro-fibres degradation rate of $d_f = 1$

**Fig. 10:**
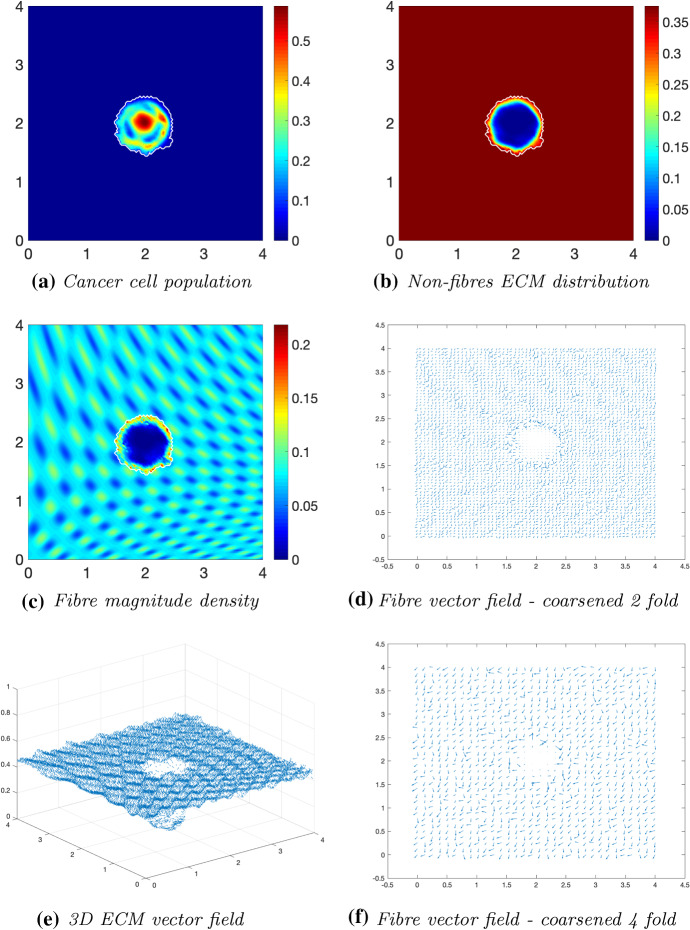
Simulations at time $50\varDelta t$ with a homogeneous distribution of the non-fibrous phase and $15\%$ heterogeneous fibres phase of the ECM with a micro-fibres degradation rate of $d_f = 1$

**Fig. 11:**
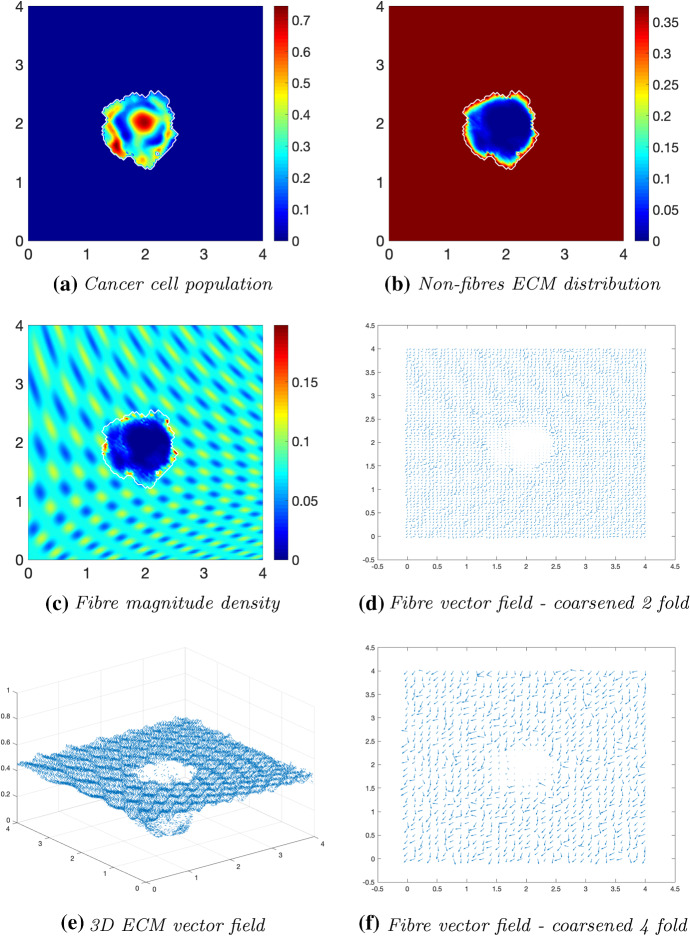
Simulations at time $75\varDelta t$ with a homogeneous distribution of the non-fibrous phase and $15\%$ heterogeneous fibres phase of the ECM with a micro-fibres degradation rate of $d_f = 1$

To investigate the effects of fibre distribution, while keeping the non-fibrous ECM phase initially homogeneous (23), we initialise the fibre distribution *F*(*x*, 0) with $15\%$, or $p=0.15$, of the heterogeneous distribution (24). The simulations at time $25 \varDelta t$ (Fig. 9) indicate the initial distribution of fibres is significant during the evolution of a tumour. The primary bulk of cancer cells have dispersed within the boundary into regions of high cell distribution (Fig. 9a), these coinciding with low-density regions of the fibrous ECM phase. The boundary of the tumour is circular with small defects in the direction of the fibre orientation (Fig. 6f). The fibre magnitude density in Fig. 9c has small regions of high fibre distributions, again pushed outwards towards the proliferating edge. Due to the heterogeneity of the fibre distributions, the fibre orientations are subject to an increased degree of realignment (Fig. 9d, f), attributed to the initially different levels of density at each macro-spatial position. The fibres are realigned towards the higher density regions, where “frenzied” groups of fibre orientations can be observed. Moving on to simulations at time $50 \varDelta t$ (Fig. 10), many of the behaviours previously observed at time $25 \varDelta t$ are magnified. The cancer bundle has increased in size and notably spread further into the areas of initially low fibre density (Fig. 10a) with the tumour boundary also increasing in size and irregularity. The fibres are being rearranged and pushed further outwards towards the tumour boundary (Fig. 10c), while simultaneously undergoing macroscopic degradation at the tissue scale (9), thus resulting in areas of very low to no fibre density. Finally, the simulations in Fig. 11 show the evolution of the tumour at time $75 \varDelta t$. The cancer cells are forming patterns within the tumour boundary (Fig. 11a), in the areas of low ECM density, and cells are migrating towards the tumour boundary collecting in high distribution bundles that more rapidly degrade the surrounding ECM. Furthermore, the cells are flooding areas where there is very low to no fibre density (Fig. 11c) and forming dense bundles of cells. This behaviour is in accordance with the conclusions presented in Shuttleworth and Trucu (2019) that cancer cells can more freely invade areas of no ECM density and will progress upon these areas first before engulfing the higher density regions.

### Increased Collagen Density

**Fig. 12:**
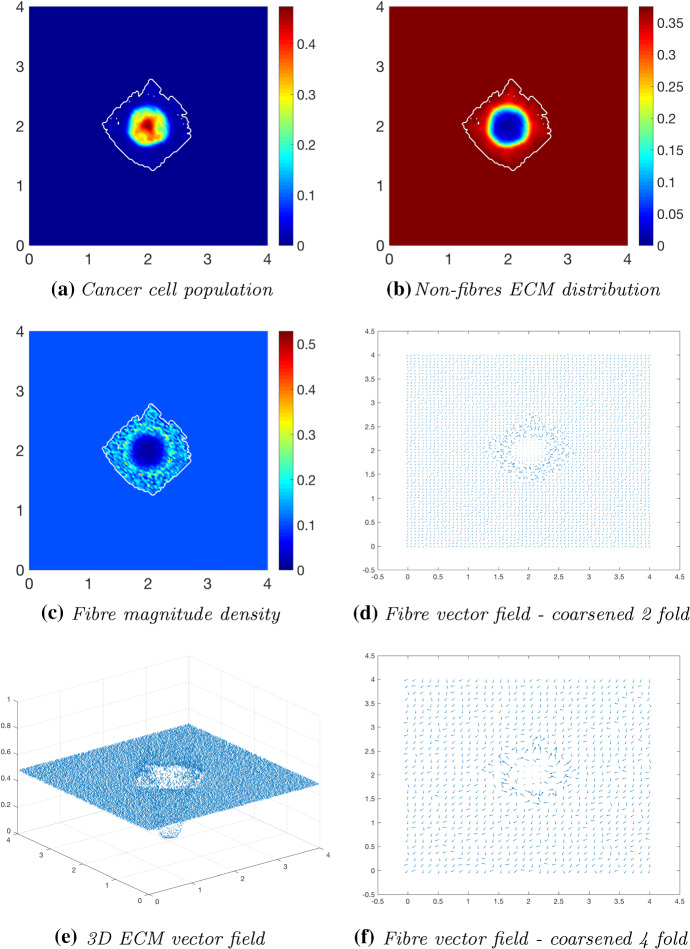
Simulations at time $25\varDelta t$ with a homogeneous distribution of the non-fibrous phase and $20\%$ homogeneous fibres phase of the ECM with a micro-fibres degradation rate of $d_f = 0.5$

**Fig. 13:**
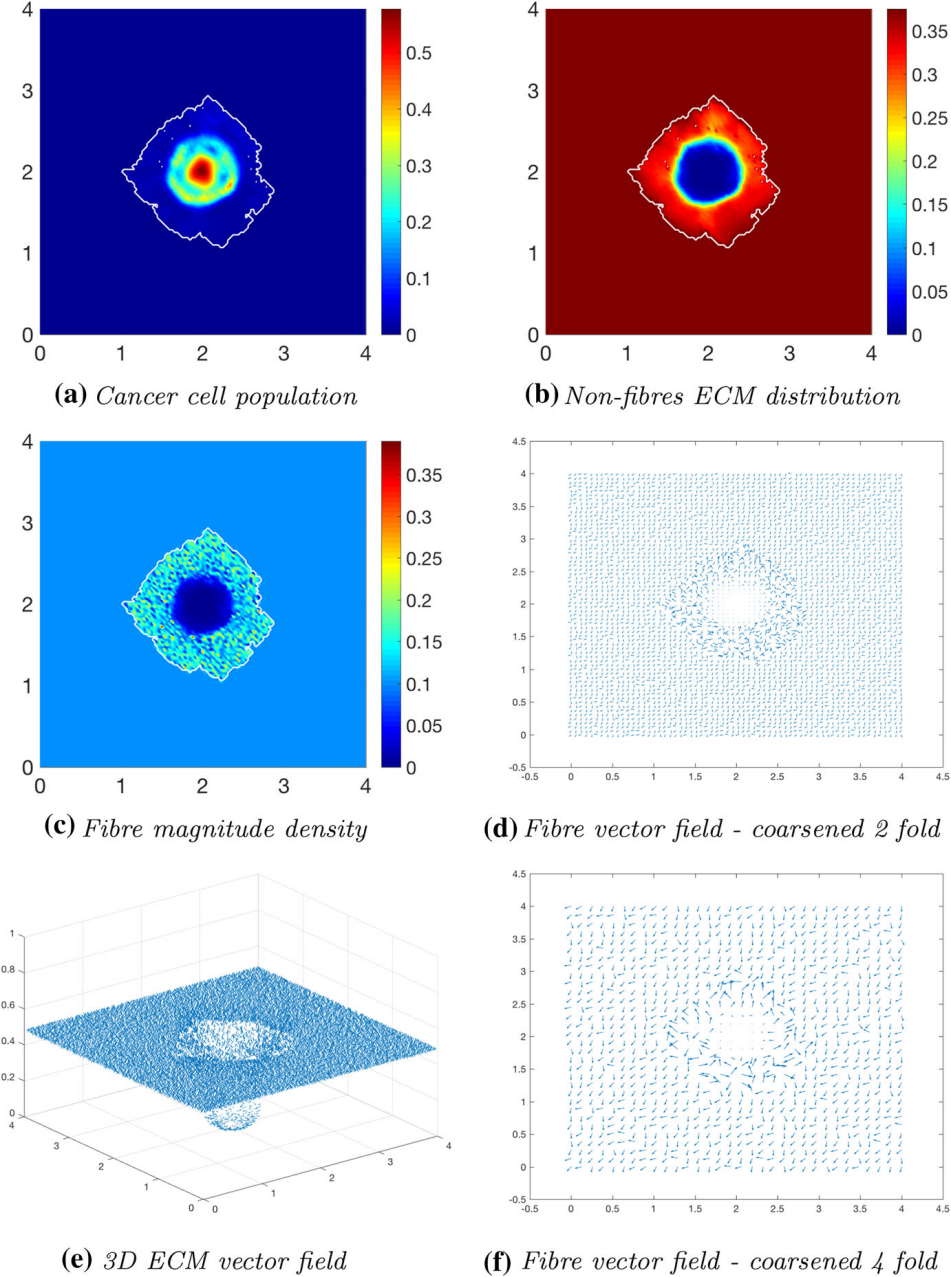
Simulations at time $50\varDelta t$ with a homogeneous distribution of the non-fibrous phase and $20\%$ homogeneous fibres phase of the ECM with a micro-fibres degradation rate of $d_f = 0.5$

**Fig. 14:**
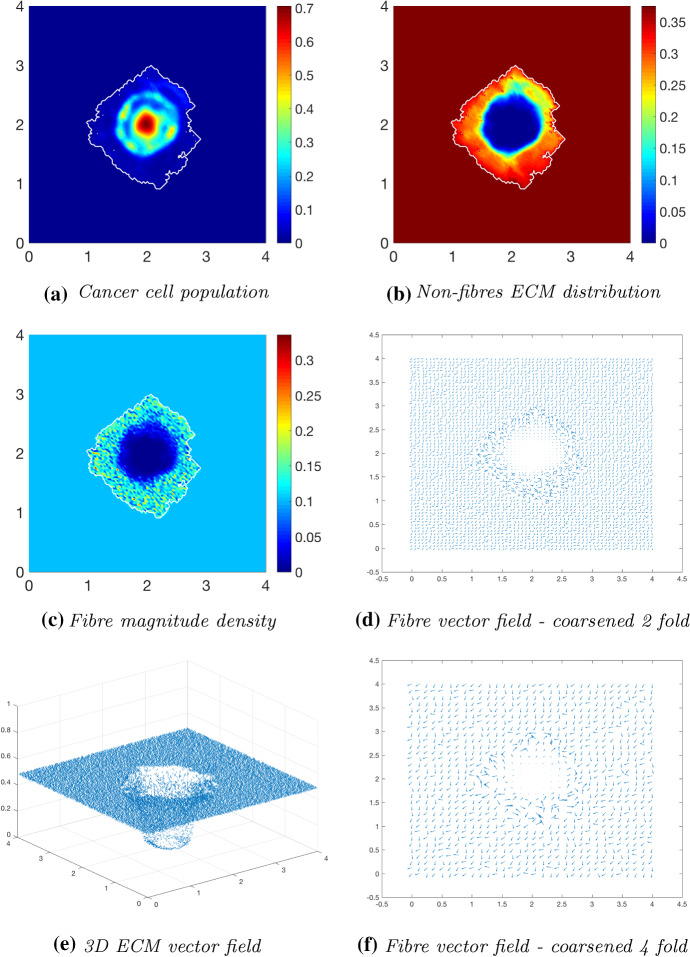
Simulations at time $75\varDelta t$ with a homogeneous distribution of the non-fibrous phase and $20\%$ homogeneous fibres phase of the ECM with a micro-fibres degradation rate of $d_f = 0.5$

We proceed by exploring the cancer dynamics within an initial $20\%$ homogeneous fibre distribution, taken as $p=0.2$ of the non-fibres ECM phase *l*(*x*, 0), and in the presence of the micro-fibre degradation rate $d_{f}=0.5$. At time $25 \varDelta t$ (Fig. 12), the non-fibrous ECM phase has been degraded by the cancer cells (Fig. 12b), with the highest degradation occurring in the regions of highest cell distribution. The tumour boundary at this stage is larger than in previous simulations, this correlating with results in Shuttleworth and Trucu (2020, 2019) where an initially higher density fibrous ECM phase resulted in an accelerated spread of the tumour. Additionally, the macroscopic fibre density (Fig. 12c) also exhibits different behaviour than in previous simulations, where a similar pattern of fibres was noted in Shuttleworth and Trucu (2020, 2019). This pattern of fibres is witnessed because the tumour boundary is expanding faster than the micro-fibres being rearranged; thus, the distributions are not found to build on the proliferating edge. Moving on to time $50 \varDelta t$, the cancer cell distribution is increasing and spreading within the tumour region (Fig. 13a). There is significant non-fibres ECM degradation stretching the entire area of the tumour (Fig. 13b), with this only becoming more pronounced at later stages (Fig. 14b). The macroscopic fibre orientations (Fig. 13d, f) display high levels of reorientation with a similar “frenzied” appearance to figures in Shuttleworth and Trucu (2020). At the final time $75 \varDelta t$ (Fig. 14), the cancer cells are dispersed further into the matrix (Fig. 14a) and noticeably in the pattern of degradation of the non-fibres ECM phase (Fig. 14b). Overall, we conclude that in the presence of a homogeneous ECM, with a high initial homogeneous fibre distribution, the cancer cells have increased opportunity for adhesion and thus they are able to easier invade the surrounding matrix, resulting in a larger tumour region and a higher level of ECM degradation.

**Fig. 15:**
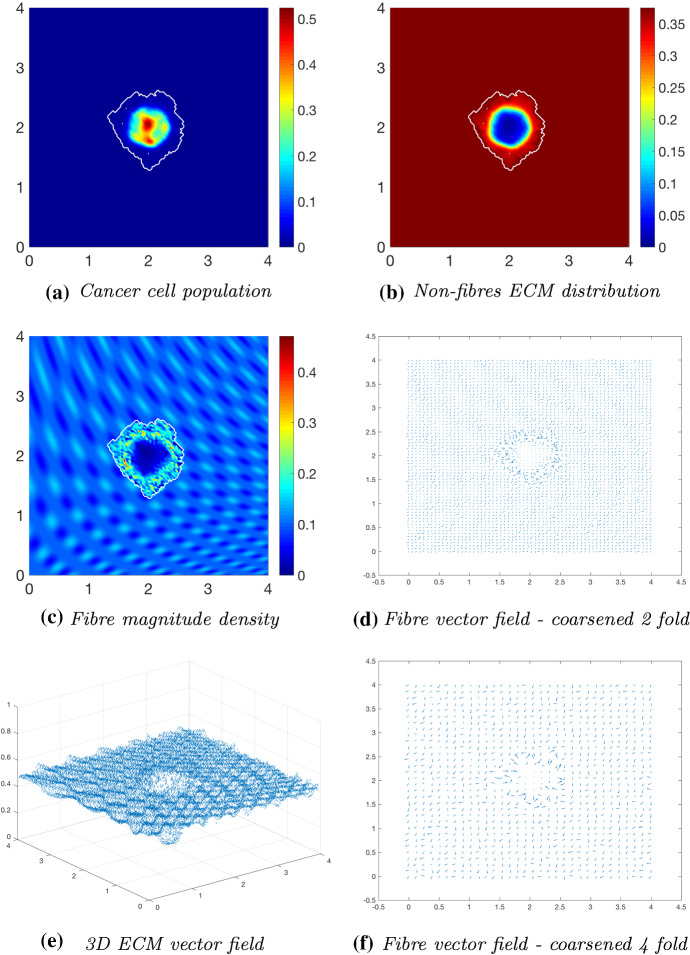
Simulations at time $25\varDelta t$ with a homogeneous distribution of the non-fibrous phase and $20\%$ homogeneous fibres phase of the ECM with a micro-fibres degradation rate of $d_f = 0.5$

**Fig. 16:**
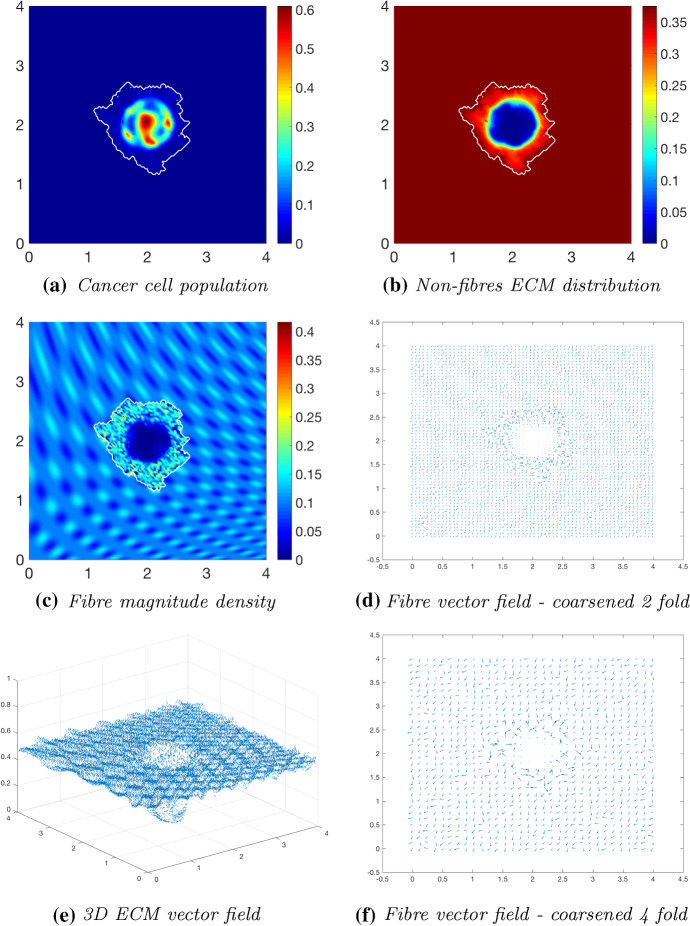
Simulations at time $50\varDelta t$ with a homogeneous distribution of the non-fibrous phase and $20\%$ homogeneous fibres phase of the ECM with a micro-fibres degradation rate of $d_f = 0.5$

**Fig. 17:**
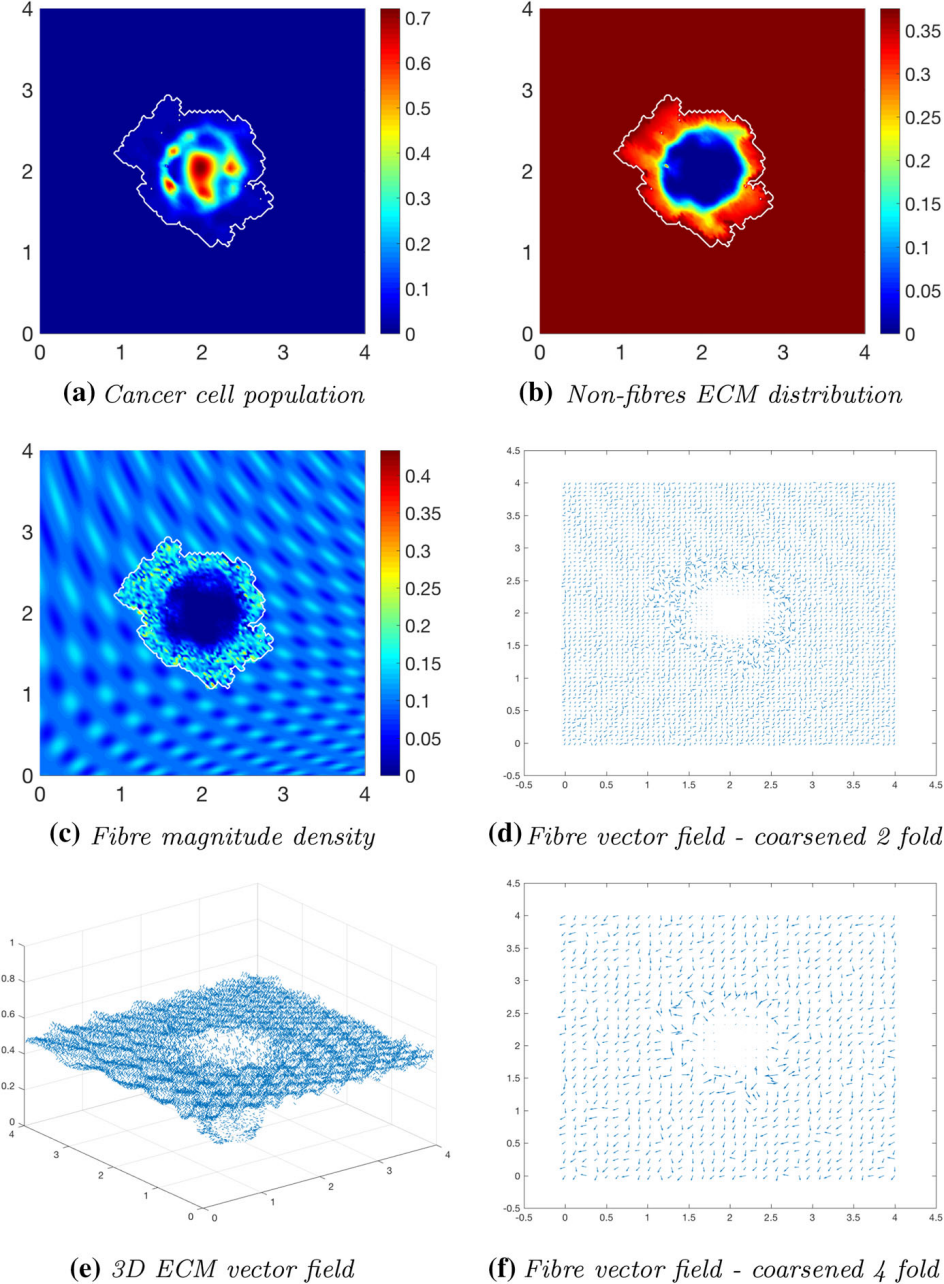
Simulations at time $75\varDelta t$ with a homogeneous distribution of the non-fibrous phase and $20\%$ homogeneous fibres phase of the ECM with a micro-fibres degradation rate of $d_f = 0.5$

We continue our investigation of cancer invasion within a collagen dense environment by exploring the multiscale model within an initially $20\%$ heterogeneous fibrous ECM phase embedded in a homogeneous non-fibrous ECM phase. Figures 15, 16 and 17 display simulation results at times $t\in \{25 \varDelta t, 50 \varDelta t, 75 \varDelta t\}$. When comparing with the results in Figs. 12, 13 and 14, the significant differences when in a homogeneous or heterogeneous fibre environment are the shape and density of the main tumour bulk and the pattern of the proliferating boundary. The tumour boundary in Fig. 15 is spreading first to the low-density regions of fibres, this behaviour consistent with previous results in Shuttleworth and Trucu (2019), where the cancer cells can more easily migrate to close-by areas of low fibre density due to the physical space available; thus, the boundary of the tumour becomes lobular as the cells migrate outwards (Fig. 15c). The main bulk of tumour cells are also exhibiting this behaviour as they have formed a high distribution region of cells in the area of lowest fibre density (Fig. 15a). The macroscopic fibre orientations have been rearranged to direct the movement of the tumour boundary, witnessed in Fig. 16 at the next time stage interval $50 \varDelta t$. The main body of the tumour has distributed the cells into several small high distribution bundles (Fig. 16a) particularly in the low-density regions of fibres (Fig. 16c). The microscopic fibres are continuously rearranged, and due to a higher rate of fibres degradation where the cancer cell distribution is highest, we observe a very low central region of fibre density with them being both degraded and pushed outwards from the central region of the tumour (Fig. 16d, f). The orientation of the fibres becomes increasingly erratic in areas of higher fibre density, witnessed in the protrusions of the tumour boundary, whereby they are orientated in opposing directions. This trait is exaggerated at final time $75 \varDelta t$ (Fig. 17), where the tumour has spread further into the ECM and the protrusions have increased in size. Furthermore, the bulk of cancer cells have become separated and formed distinct regions within the tumour boundary. When comparing the cancer cell distribution and the macro-fibres density (Fig. 17a, c), respectively, it can be noted that the cancer cells have bypassed the higher regions of fibres and formed bundles of cells around these areas. This behaviour can also be seen in the homogeneous case; however, it is a more prominent feature when in the presence of a heterogeneous fibrous ECM phase. In conclusion, in the presence of an initially high fibre ECM density, tumour progression is accelerated and encourages a more aggressively spreading tumour, in both the cases of either a homogeneous or heterogeneous fibre distribution. Additionally, when the initial fibre density is high, the boundary of the tumour spreads further away from the main body of the tumour at a rate which the cells cannot maintain. Hence, the main body of the tumour stays centralised, compared to a lower initial fibre density, whereby the cancer cells spread at a consistent rate within the tumour region and stay close to the boundary.

### Invasion Patterns of a Heterotypic Cancer Cell Population

To conclude our exploration of cancer invasion within a heterogeneous microenvironment, we focus now on the invasion patterns of a heterotypic cell population using the two cell sub-populations macro-dynamics introduced in Shuttleworth and Trucu (2018) and expanded upon in Shuttleworth and Trucu (2020). The macro-dynamics of the two-cell sub-populations are similar in flavour and can be mathematically expressed as

25 $$\begin{aligned} \frac{\partial c_1}{\partial t}&= \nabla \cdot [D_{1} \nabla c_1 - c_1 \mathcal {A}_{1}(x,t,\mathbf{u} (t,\cdot ),\theta _{f}(\cdot ,t))] +\mu _{1}c_1(1-\rho (\mathbf{u} )) - M_{c}(\mathbf{u} ,t)c_1, \end{aligned}$$

26 $$\begin{aligned} \frac{\partial c_2}{\partial t}&= \nabla \cdot [D_{2} \nabla c_2 - c_2 \mathcal {A}_{2}(x,t,\mathbf{u} (t,\cdot ),\theta _{f}(\cdot ,t))] +\mu _{2}c_2(1-\rho (\mathbf{u} )) + M_{c}(\mathbf{u} ,t)c_1, \end{aligned}$$

where the individual terms retain their exact meaning from Sect. 2.2, while $M_{c}(\mathbf{u} ,t)$ represents the mutation of cells from cell population $c_{1}$ to cell sub-population $c_{2}$, defined in Shuttleworth and Trucu (2020),

$$\begin{aligned} M_{c}(x,t):= \left\{ \begin{array}{ll} \frac{\text {exp}\left( \frac{-1}{\kappa ^{2}-(1-v(x,t))^{2}}\right) }{\text {exp}\left( \frac{1}{\kappa ^{2}}\right) } \cdot H(t-t_m) &{}\quad \text {if} \ 1-\kappa<v(x,t)<1, \\ 0, &{}\quad \text { otherwise }. \end{array} \right. \end{aligned}$$

Here, the mutations are considered to be dependent on the underlying fibre densities, with $\kappa$ representing a certain level of ECM beyond which mutations can occur, $H(\cdot )$ being the usual Heaviside function, with $t_m$ being the time at which mutations begin. Furthermore, we consider both cancer cells sub-populations to contribute to the source of MMPs on the tumour boundary; hence, the microscopic source term (13) will be readdressed as

27 $$\begin{aligned} \begin{aligned} 1. \quad&g_{\epsilon Y}(y,\tau ) = \frac{\int \limits _\mathbf{B (y,\gamma )\cap \varOmega (t_0)} (\alpha _{1} c_{1}(x,t_0 + \tau ) + \alpha _{2} c_{2}(x,t_0 + \tau )) \ \widetilde{F}(x,t_0 + \tau ) \ \mathrm{d}x}{\widetilde{F} \cdot \lambda (\mathbf{B} (y,\gamma )\cap \varOmega (t_0))}, \\&\qquad \qquad \qquad \qquad \qquad \qquad \qquad \qquad \qquad \qquad \qquad \qquad \qquad \qquad y \in \epsilon Y \cap \varOmega (t_0), \\ 2. \quad&g_{\epsilon Y}(y,\tau ) = 0,\quad y \in \epsilon Y {\setminus } \big ( \varOmega (t_0)+\{ y \in Y| \ ||y||_2 < \gamma \}), \end{aligned} \end{aligned}$$

where $\alpha _{1}, \alpha _{2}$ are the MMP secretion rates for cell populations $c_{1}$ and $c_{2}$, respectively, $\widetilde{F}(x,t_0 + \tau ):=1+F(x,t)$ and $\widetilde{F} \cdot \lambda$ are defined as in Sect. 3. Using parameter set $\varSigma _{1}$ from “Appendix C”, the micro-fibre degradation rate $d_{f}=1$ and the cell adhesion matrices

$$\begin{aligned} \mathbf{S} _{max}= \left( \begin{array}{cc} 0.5 &{} 0 \\ 0 &{} 0.3 \end{array} \right) \quad \text {and} \quad \mathbf{S} _{cF}= \left( \begin{array}{cc} 0.1 &{}\quad 0 \\ 0 &{}\quad 0.2 \end{array} \right) , \quad \mathbf{S} _{cl}= \left( \begin{array}{cc} 0.05 &{}\quad 0 \\ 0 &{}\quad 0.05 \end{array} \right) , \end{aligned}$$

**Fig. 18:**
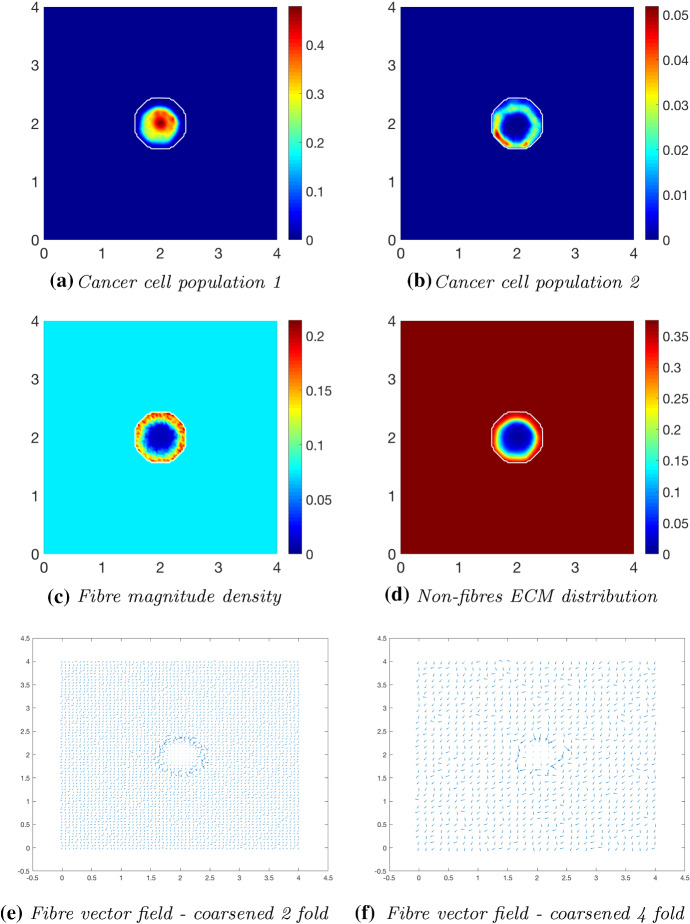
Simulations at time $25\varDelta t$ with a homogeneous distribution of the non-fibrous phase and $15\%$ homogeneous fibres phase of the ECM with a micro-fibres degradation rate of $d_f = 1$

**Fig. 19:**
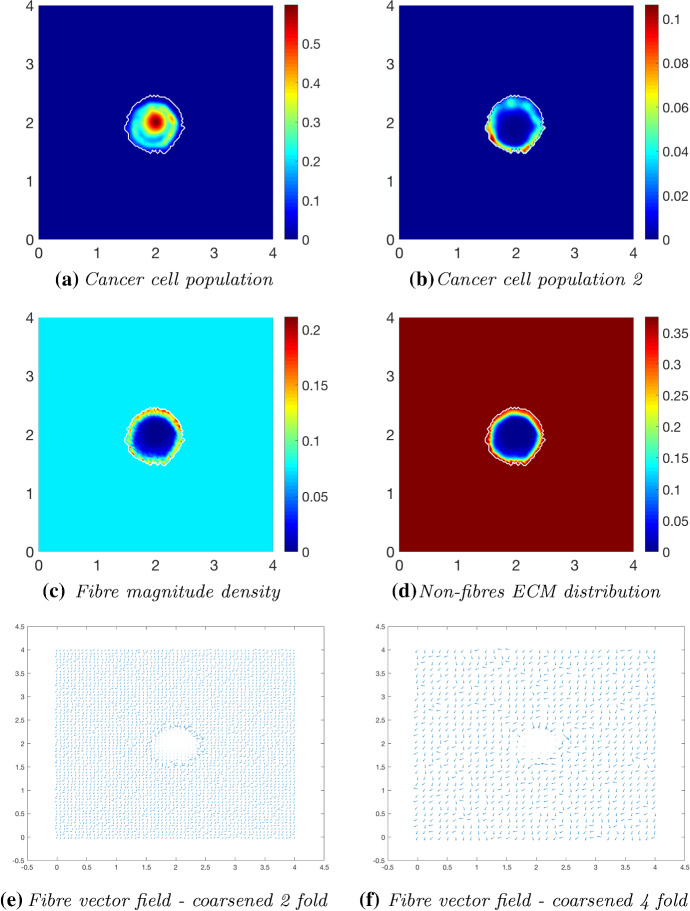
Simulations at time $50\varDelta t$ with a homogeneous distribution of the non-fibrous phase and $15\%$ homogeneous fibres phase of the ECM with a micro-fibres degradation rate of $d_f = 1$

**Fig. 20:**
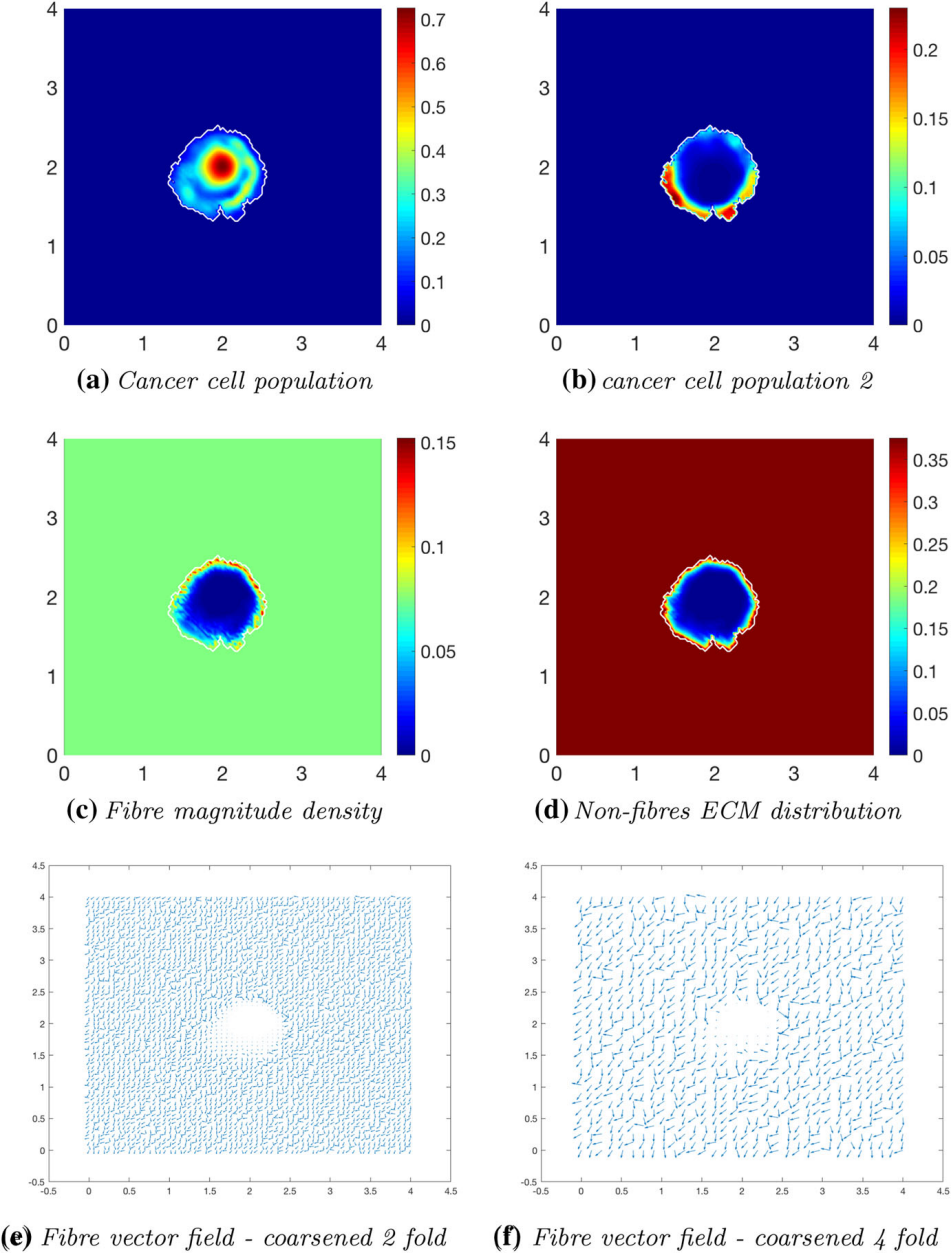
Simulations at time $75\varDelta t$ with a homogeneous distribution of the non-fibrous phase and $15\%$ homogeneous fibres phase of the ECM with a micro-fibres degradation rate of $d_f = 1$

**Fig. 21:**
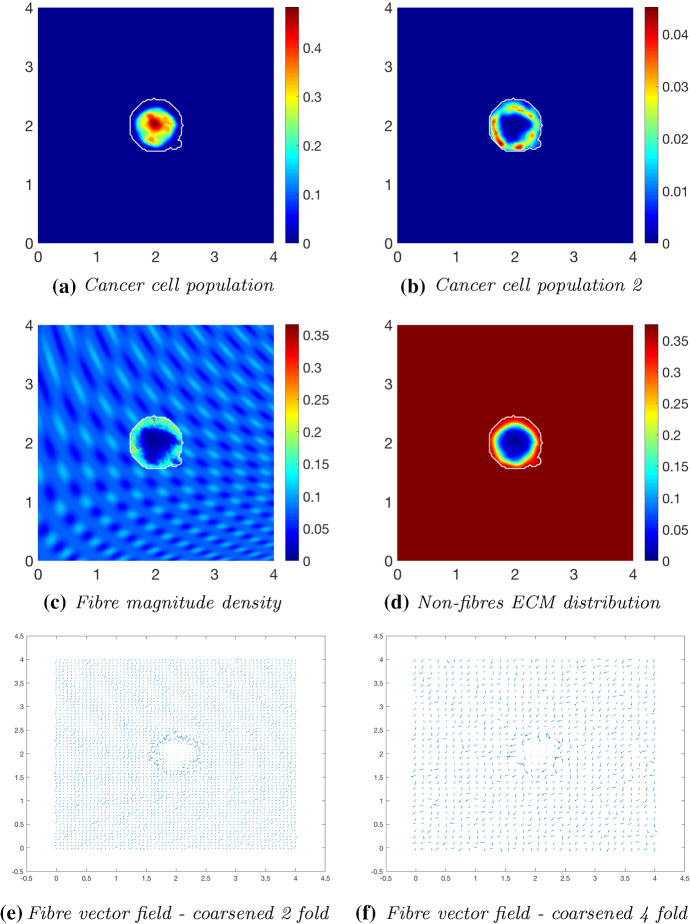
Simulations at time $25\varDelta t$ with a homogeneous distribution of the non-fibrous phase and $15\%$ heterogeneous fibres phase of the ECM with a micro-fibrous degradation rate of $d_f = 1$

**Fig. 22:**
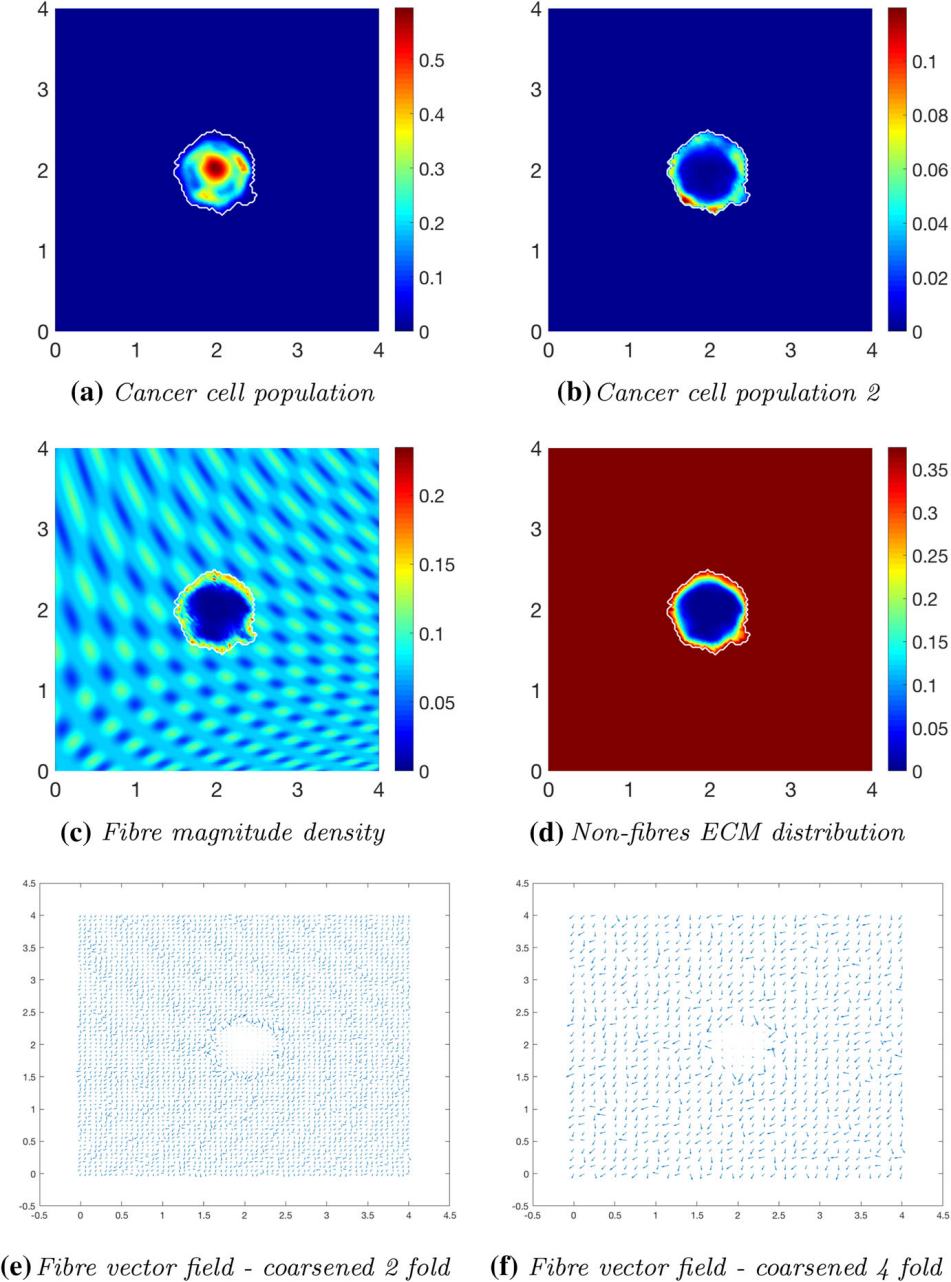
Simulations at time $50\varDelta t$ with a homogeneous distribution of the non-fibrous phase and $15\%$ heterogeneous fibrous phase of the ECM with a micro-fibres degradation rate of $d_f = 1$

**Fig. 23:**
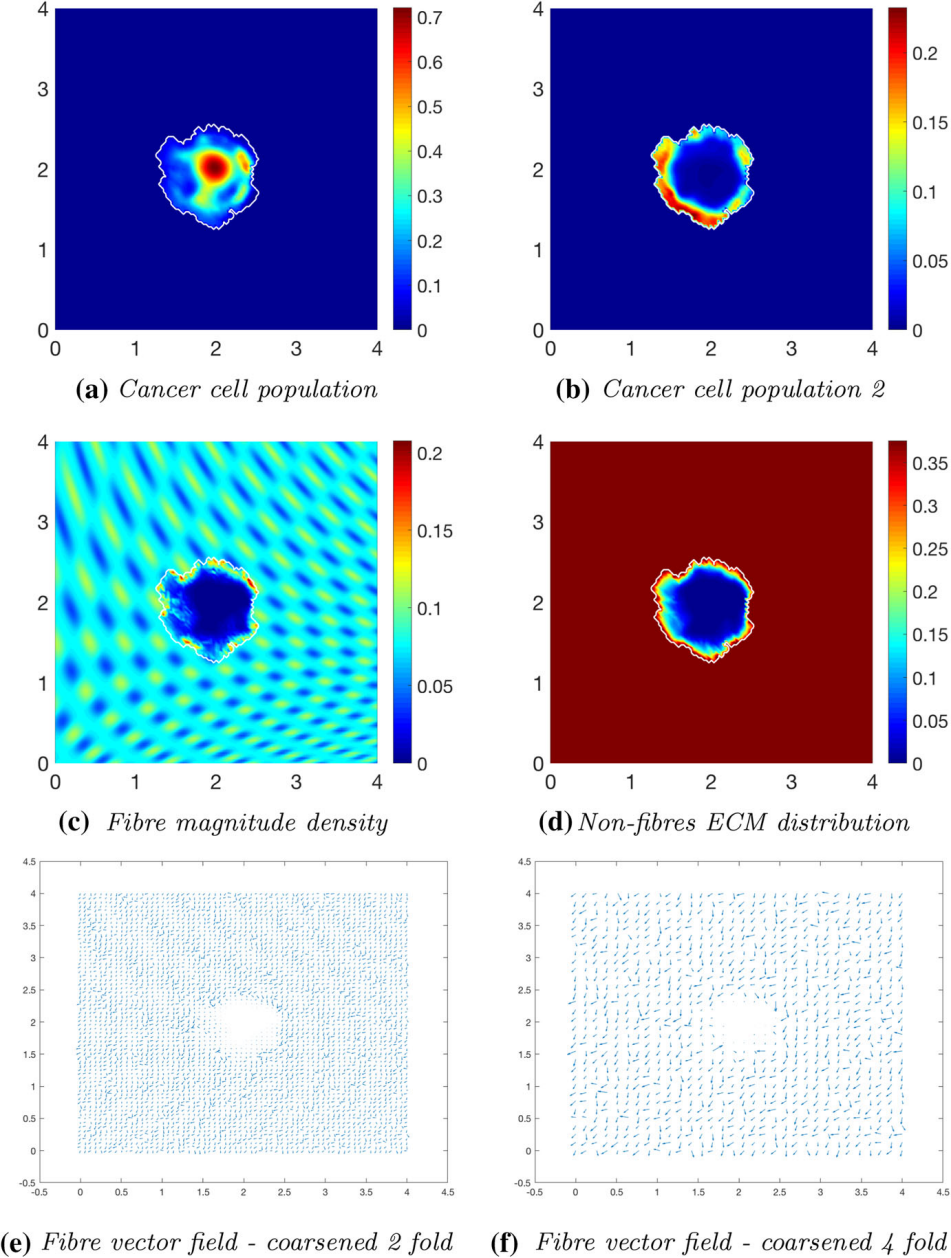
Simulations at time $75\varDelta t$ with a homogeneous distribution of the non-fibrous phase and $15\%$ heterogeneous fibrous phase of the ECM with a micro-fibres degradation rate of $d_f = 1$

in Figs. 18, 19, 20, 21, 22 and 23, we present the computational results for the evolution of: (a) the primary cancer cell sub-population; (b) the secondary cell sub-population; (c) the fibre magnitude density; (d) the non-fibres ECM distribution; and of the vector field of orientated fibres at two different resolutions, namely: (e) coarsened twice; and (f) coarsened fourfold. Considering an initially homogeneous fibrous ECM phase, when comparing these results directly with the results in Shuttleworth and Trucu (2020), we observe no differences between simulations performed in the absence of micro-fibre degradation at the tumour interface at the first interval $25 \varDelta t$, as shown in Fig. 18, with the fibre orientations consistent between the results (Fig. 18e, f). Proceeding to later times , $50 \varDelta t$ and $75 \varDelta t$, shown in Figs. 19 and 20, respectively, we begin to witness changes, whereby the boundary of the tumour is visibly smaller than in previous results, thus implying a slower progression of the tumour. The macroscopic fibre orientations are aligned differently in Fig. 19e, f, where the fibres are directed inwards to the centre of the tumour, confining the tumour to the centre of the domain (Fig. 18a). Moving on to the final stage (Fig. 20), the tumour region is smaller than in previous results in Shuttleworth and Trucu (2020). The bulk of the cancer cells stick closely to the tumour boundary and both the fibrous and non-fibrous ECM phases have undergone a higher level of degradation within the tumour region.

Finally, to further our understanding of the effects of micro-fibre degradation at the tumour interface, we explore the evolution of a heterotypic cell population within a heterogeneous fibrous ECM phase, while the non-fibrous ECM phase remains homogeneous, as shown in Figs. 20 and 21. As with an initially homogeneous fibre density, at time $25 \varDelta t$ (Fig. 21), there is very little difference when compared to simulations presented in Shuttleworth and Trucu (2020). Moving on to later stages, it is obvious that the process of boundary micro-fibre degradation causes a slower rate of tumour progression. The tumour region is considerably smaller in Figs. 22 and 23 when compared with previous results (Shuttleworth and Trucu 2020). The bulk of tumour cells display a similar pattern, however much closer to the tumour boundary, particularly cancer cell population $c_{2}$ where the cells have formed high-distribution bundles at the leading edge. Much like in the presence of a homogeneous fibre density, both the fibrous and non-fibrous ECM phases have been subject to a higher level of degradation in the tumour region. This is attributed to the micro-fibre degradation at the tumour boundary; as degradation continuously occurs as the boundary expands, we witness a lower fibre density as the tumour evolves. It can be concluded from these simulations and comparisons with previous results that the process of micro-fibre degradation at the tumour interface is disadvantageous to tumour progression, inhibiting the full invasive capabilities of the tumour. This is due to the lower levels of fibre density at the tumour interface; as degradation of the micro-fibres occurs both inside and within the peritumoural region of the tumour, the cell–fibre adhesion rate is reduced in line with low fibre levels; therefore, the cancer cells do not have the same opportunities for adhesion and thus their migration is greatly reduced.

## Discussion

In this paper, we have presented an integrated two-part multiscale model of cancer invasion, which builds on the approach introduced in Shuttleworth and Trucu (2019) and extends that to capture explicitly the dynamic cell-scale interaction between the MDE boundary micro-dynamics and the peritumoural mass distribution of micro-fibres.

Structured largely similar to the modelling framework introduced in Shuttleworth and Trucu (2019), the model proposed here combines two multiscale systems that share the same tissue-scale (macro-scale) dynamics while having separate cell-scale (micro-scale) processes that are simultaneously connected via two double-feedback loops. Specifically, while this new modelling framework inherits completely the multiscale dynamics of naturally oriented ECM fibres (induced by the mass distribution of micro-fibres) occurring on the topological closure of the invading tumour (and including the dynamic rearrangement of fibres under the incidence of the macro-scale flux of cancer cells), this shares its macro-dynamics with a significantly extended multiscale moving-boundary modelling for the proteolytic dynamics at the tumour invasive edge that explicitly considers the interaction with the peritumoural fibres.

This model expands and takes forward both the initial multiscale moving-boundary framework introduced in Trucu et al. (2013) and its further development into the two-part multiscale modelling introduced in Shuttleworth and Trucu (2019) by bringing in and exploring the cell-scale interactions between the cross-interface diffusion of MMPs and the micro-fibre distributions in the peritumoural region, with direct impact upon microscopic peritumoural degradation of micro-fibres that results in a continuously altered macroscopic vector field of oriented ECM fibres at the tumour boundary. Moreover, these altered peritumoural ECM fibres have major relevance within the macroscopic dynamics of the cancer cells as this affects the cell–fibre adhesion properties at the leading edge of the tumour, impacting this way not only the tumour mechanics close to the tumour interface but the entire tissue-scale dynamics of the tumour.

To that end, we first explore mathematically the positive feedback that the macroscopic distribution of ECM fibres close to the tumour interface has upon the emerging cell-scale source of MMP-2 for the cross-interface micro-dynamics that MMP-2 exercises at the invading edge of the tumour. Specifically, in this new formulation, we are able to capture the enhanced sources of MMP-2 in a relevant cell-scale neighbourhood which are enabled non-locally by the presence of elevated distributions of ECM fibres within neighbouring active regions from within the outer proliferating rim of the tumour where cancer cells arrive during their macro-dynamics and produce MMPs.

Further, in the presence of the cell-scale MMP-2 source induced by the macro-dynamics, a cross-interface diffusion of MMP-2 occurs at the invasive edge of the tumour. However, as the MMP-2 find it easier to diverge along their gradient directions in regions with lower micro-fibrous levels, by accounting for the presence micro-scale mass distribution, we finally obtain that the diffusion rate of this diffusive molecular transport process of MMP-2 naturally depends on the micro-fibrous density. Thus, this cell-scale MMP-2 dynamics focuses the cross-interface molecular transport towards the regions of lower mass distributions of micro-fibres, taking *on-the-fly* advantage on the potential “micro-fibres valleys” created by the multiscale dynamic rearrangement of fibres induced by the macro-scale flux of cancer cells, which was derived and explored with full details in Shuttleworth and Trucu (2019). Finally, in this work, for the molecular MMP-2 micro-dynamics (14), we only considered the isotropic formulation but with the diffusion coefficient being dependent on the amount of micro-fibres that manifest a local attenuation effect upon the spatiotemporal diffusive transport of MMP-2. However, an extension of this will be to consider the full anisotropic case enabled by the micro-scale spatial distribution of micro-fibres, which will also lead to a new formulation of equation (14), but that will be part of a future work.

Since the MMP-2 cross-interface molecular transport leads to peritumoural micro-fibres degradation at the micro-scale, we explored this degradation explicitly at the cell scale, remarking here at the same time that this complements the previous modelling framework introduced and discussed in Shuttleworth and Trucu (2019) where the fibres degradation was only considered on the bulk of the tumour at the macro-scale. To that end, we considered the correlation between the micro-fibres degradation and the incidence angle that the MMP-2 molecular flux makes with the regions of significant levels of micro-fibres distributed with any given $\epsilon Y$ from the covering bundle of $\{\epsilon Y\}_{\epsilon Y\in \mathcal {P}(t)}$ boundary micro-domains. This enabled us to derive mathematically a micro-fibres degradation law occurring on each fibre micro-domain $\delta Y(x)$ that has non-empty intersection with at least one of the boundary micro-domains $\epsilon Y$, in which maximum fibre degradation occurs when the angle between the fibres and MMPs flux is perpendicular, while the degradation decreases with increasing alignment of the fibres with the direction of the MMP-2 molecular flux. This suggests that the highly aligned collagen fibrils will act as a pathway for invasion rather than a barrier against it, this being consistent with the biological evidence presented in Provenzano et al. (2006). Finally, this degradation of peritumoural mass distribution micro-fibres at the cell scale is continuously in time translated back at macro-scale, having a natural and major impact upon *on-the-fly* changes in the orientation and magnitudes of macro-scale ECM fibres from the peritumoural region.

This new modelling framework has been explored in several scenarios, within both a homogeneous and heterogeneous initial distribution of fibres, and varying the initial ratio of macroscopic fibre distribution in relation to the non-fibrous ECM phase. The non-fibrous ECM phase was kept as a homogeneous density throughout the paper for the purpose of exploring only the influence of ECM fibres during cancer invasion. These scenarios were explored through randomly allocated distributions of micro-fibres over the fibres micro-domain, as considered already in Shuttleworth and Trucu (2020) and defined in “Appendix B”.

We explored the differences between a homogeneous and heterogeneous initial distribution of fibres where we varied the initial percentage of fibrous density from $15\%$ to $20\%$, as well as investigating the morphology of a heterotypic cancer cell population whose macroscopic dynamics were developed in Shuttleworth and Trucu (2020). We conclude from these simulations that a heterogeneous distribution of fibres induces a more lobular, fingering pattern of the tumour boundary, and an increase in initial fibre density promotes a more aggressive tumour spreading further into the surrounding tissue, a behaviour which is mirrored in the biological experiments performed in Provenzano et al. (2008). These remarks are in line with previous results in Shuttleworth and Trucu (2019, 2020) where the same conclusions are drawn regarding the initial condition of the fibrous ECM phase. Finally, it is perhaps worth mentioning here that, while it was not included in this paper, a sensitivity analysis with respect to the percentage of initial fibres within the ECM initial conditions of the model has been carried out and was presented (Shuttleworth and Trucu 2019). That sensitivity analysis has shown that, in the presence of a homogeneous non-fibrous ECM phase, by gradually reducing the level ECM fibres towards very small magnitudes within ECM, we observe a consistent behaviour of the model that gets correspondingly closer to the one obtained when no fibres were considered in the ECM.

The simulations performed in this paper exhibit an overall larger tumour spread than the simulations in Shuttleworth and Trucu (2019), implying that the degradation of fibres at the tumour interface promotes tumour invasion. The final simulations, which consider the invasive behaviour of a heterotypic cell population, suggest that although the cancer cells migrate more easily into low-density regions of ECM, at the tumour interface a lack of fibre density is detrimental to the progression of the tumour. The low levels of fibre density inhibit the migration of cancer cells and thus the movement of the tumour boundary by reducing the opportunities for cell–fibre adhesion. In general, we conclude that the invasion of a heterotypic cancer cell population is accelerated in the presence of a high fibrous ECM density; however, within a low fibrous ECM, the tumour undergoes slower progression and the bulk of the cancer cells remain closer to the gradually expanding tumour boundary.

Looking forward, to advance with this model, we would look to investigate the full MT1-MMP/MMP-2 cascade, namely to include within the model the tissue inhibitor of matrix-metalloproteinases-2, TIMP-2, a key molecule required for the activation of pro-MMP-2 (Seiki and Yana 2003), considering the role and regulation of these molecules and the resulting effects on peritumoural tissue degradation. Additionally, further exploration of the fibres network and its structure would permit better, more realistic modelling of human tissue, allowing the model to be compared with current biological experiments, for example, with the experiments performed in Provenzano et al. (2008) that investigate the effects of increasing collagen density in the surrounding matrix.

## The Mollifier $\psi _{\gamma }$

The standard mollifier $\psi _{\gamma }:\mathbb {R}^{N}\rightarrow \mathbb {R}_{+}$ (which was used also in Shuttleworth and Trucu (2019) Trucu et al. (2013)) is defined as usual, namely

$$\begin{aligned} \psi _{\gamma }(x):=\frac{1}{\gamma ^{N}}\psi \big (\frac{x}{\gamma }\big ), \end{aligned}$$

where $\psi$ is the smooth compact support function given by

$$\begin{aligned} \psi (x):= \left\{ \begin{array}{lll} \frac{exp\frac{1}{\parallel x \parallel ^{2}_{{2}}-1}}{\int \limits _{\mathbf {B}(0,1)}\exp \frac{1}{\parallel z \parallel ^{2}_{{2}}-1}\mathrm{d}z},&{} \quad \hbox {if} &{} x\in \mathbf {B}(0,1),\\ 0, &{} \quad \hbox {if} &{} x\not \in \mathbf {B}(0,1). \end{array} \right. \end{aligned}$$

## Microscopic Fibre Domains

For the fibres initial conditions,, on the micro-domains $\delta Y(x)$, we consider a family of five distinctive micro-fibres patterns, $\{P^{1}_{i}\}_{i\in J}$, which are defined by the union of paths $P^{1}_{i}=\bigcup _{j=1\ldots 5} h^{1}_{i,j}$, which are given as follows.

*For the family of fibre paths*$P^{1}$*, we have:*

$$\begin{aligned} h^{1}_{1,1}: z_{1}= & {} z_{2}; \quad h^{1}_{1,2}: z_{1}=\frac{1}{2}; \quad h^{1}_{1,3}: z_{1}=\frac{1}{5}; \quad h^{1}_{1,4}: z_{2}=\frac{2}{5};\quad h^{1}_{1,5}: z_{2}=\frac{4}{5}. \\ h^{1}_{2,1}: z_{1}= & {} z_{2}; \quad h^{1}_{2,2}: z_{1}=\frac{1}{2}; \quad h^{1}_{2,3}: z_{1}=\frac{1}{5}; \quad h^{1}_{2,4}: z_{2}=\frac{2}{5};\quad h^{1}_{2,5}: z_{2}=\frac{1}{10}. \\ h^{1}_{3,1}: z_{1}= & {} z_{2}; \quad h^{1}_{3,2}: z_{1}=\frac{1}{10}; \quad h^{1}_{3,3}: z_{1}=\frac{9}{10}; \quad h^{1}_{3,4}: z_{2}=\frac{2}{5};\quad h^{1}_{3,5}: z_{2}=\frac{1}{10}. \\ h^{1}_{4,1}: z_{1}= & {} z_{2}; \quad h^{1}_{4,2}: z_{1}=\frac{1}{10}; \quad h^{1}_{4,3}: z_{1}=\frac{3}{10}; \quad h^{1}_{4,4}: z_{2}=\frac{2}{5};\quad h^{1}_{4,5}: z_{2}=\frac{1}{10}. \\ h^{1}_{5,1}: z_{1}= & {} z_{2}; \quad h^{1}_{5,2}: z_{1}=\frac{4}{5}; \quad h^{1}_{5,3}: z_{1}=\frac{3}{10}; \quad h^{1}_{5,4}: z_{2}=\frac{1}{10};\quad h^{1}_{5,5}: z_{2}=\frac{7}{10}. \end{aligned}$$

For this family of fibre paths $P^{1}$, as described in Shuttleworth and Trucu (2019), the micro-scale fibrous pattern within each micro-domain $\delta Y(x)$ is given as

28 $$\begin{aligned} f(z,t):=\sum \limits _{j=1}^{5}\psi _{h^{l}_{i,j}}(z)(\chi _{{(\delta -2\gamma _{{0}})Y(x)}}*\psi _{\gamma _{{0}}})(z) \end{aligned}$$

where $\{\psi _{h^{l}_{i,j}}\}_{i,j=1\ldots 5}$ are smooth compact support functions of the form

29 $$\begin{aligned}&\psi _{h_{j}}: \delta Y(x) \rightarrow \mathbb {R}\nonumber \\&\text { defined as follows: }\nonumber \\&Case\,1: \text { if } h^{l}_{i,j} \text { is not parallel to } z_{1}- \text { axis } \nonumber \\&\text { (i.e. } h^{l}_{i,j} \text { is identified as the graph of a function of } z_{2})\nonumber \\&\text { we have: }\nonumber \\&\psi _{h^{l}_{i,j}}(z_1,z_2):= \left\{ \begin{array}{ll} C_{h^{l}_{i,j}} e^{-{\frac{1}{r^2-(h_{j}(z_2) - z_1)^2}}}, &{}\quad \text { if } \ z_1 \in [h^{l}_{i,j}(z_2)-r, h^{l}_{i,j}(z_2)+r], \\ 0, &{}\quad \text { if } \ z_1 \not \in [h^{l}_{i,j}(z_2)-r, h^{l}_{i,j}(z_2)+r]; \end{array} \right. \nonumber \\&Case\,2: \text { if } h^{l}_{i,j} \text { is parallel to } z_{1}-\text { axis }\nonumber \\&\text { (i.e. } h^{l}_{i,j} \text { is identified as the graph of a constant function of } z_{1})\nonumber \\&\text { we have: }\nonumber \\&\psi _{h^{l}_{i,j}}(z_1,z_2):= \left\{ \begin{array}{ll} C_{h^{l}_{i,j}} e^{-{\frac{1}{r^2-(h^{l}_{i,j}(z_1) - z_2)^2}}}, &{}\quad \text { if } \ z_2 \in [h^{l}_{i,j}(z_1)-r, h^{l}_{i,j}(z_1)+r], \\ 0, &{}\quad \text { if } \ z_2 \not \in [h^{l}_{i,j}(z_1)-r, h^{l}_{i,j}(z_1)+r]. \end{array} \right. \end{aligned}$$

Here, $r>0$ is the width of the micro-fibres and $C_{h^{l}_{i,j}}$ are constants that determine the maximum height of $\psi _{h^{l}_{i,j}}$ along the smooth paths $\{h^{l}_{i,j}\}_{i,j=1\ldots 5}$ in $\delta Y(x)$. Finally, $\psi _{\gamma }$ is the standard mollifier defined in “Appendix A”, with $\gamma _{{0}}= h/16$.

Finally, the initial spatial configuration of the pattern of macroscopic ECM fibrous phase is selected according to a randomly generated matrix of labels $A=(a_{i,j})_{i,j=1\ldots n}$ corresponding to the entire $n\times n$ grid discretising *Y*, in which the entries $a_{i,j}$ are allocated values randomly selected from the set of configuration labels $\{1,2,3,4,5\}$ that will dictate the choice of micro-fibrous pattern among those described above that will be assigned to the micro-domains $\delta Y(j\varDelta x, i\varDelta y)$, for all $i,j=1\ldots n$.

## Table for the Parameter Set $\varSigma _{1}$

Here, we present a Table 1 for the parameter set $\varSigma _{1}$.

**Table 1:** Parameters in $\varSigma _{1}$

Parameter	Value	Description	References
$D_1$	$3.5 \times 10^{-4}$	Diffusion coeff. for cell population $c_{1}$	Domschke et al. (2014)
$D_2$	$7\times 10^{-4}$	Diffusion coeff. for cell population $c_{2}$	Domschke et al. (2014)
$D_m$	$10^{-3}$	Diffusion coeff. for MDEs	Estimated
$\mu _1$	0.25	Proliferation coeff. for cell population $c_{1}$	Domschke et al. (2014)
$\mu _2$	0.25	Proliferation coeff. for cell population $c_{2}$	Domschke et al. (2014)
$\gamma _{1}$	2	Non-fibrous ECM degradation coeff.	Shuttleworth and Trucu (2019)
$\gamma _{2}$	1.5	Macroscopic fibre degradation coeff.	Peng et al. (2017)
$\alpha _{1}$	1	MDE secretion rate of $c_{1}$	Estimated
$\alpha _{2}$	1.5	MDE secretion rate of $c_{2}$	Estimated
*R*	0.15	Sensing radius	Shuttleworth and Trucu (2019)
*r*	0.0016	Width of micro-fibres	Shuttleworth and Trucu (2019)
$f_{\text {max}}$	0.6360	Max. micro-density of fibres	Shuttleworth and Trucu (2019)
*p*	0.15-0.2	Percentage of non-fibrous ECM	Estimated
*h*	0.03125	Macro-scale spatial discretisation size	Trucu et al. (2013)
$\epsilon$	0.0625	Size of micro-domain $\epsilon Y$	Trucu et al. (2013)
$\delta$	0.03125	Size of micro-domain $\delta Y$	Shuttleworth and Trucu (2019)
